# Constitutive down‐regulation of liguleless alleles in sorghum drives increased productivity and water use efficiency

**DOI:** 10.1111/pbi.70150

**Published:** 2025-06-01

**Authors:** Nikhil Jaikumar, Truyen Quach, Zhengxiang Ge, Natalya Nersesian, Shirley J. Sato, Scott M. McCoy, Ming Guo, Andrew D.B. Leakey, Stephen P. Long, Tom Elmo Clemente

**Affiliations:** ^1^ Carl R. Woese Institute for Genomic Biology University of Illinois at Urbana‐Champaign Urbana Illinois USA; ^2^ DOE Center for Advanced Bioenergy and Bioproducts Innovation Urbana Illinois USA; ^3^ Department of Agronomy & Horticulture University of Nebraska‐Lincoln Lincoln Nebraska USA; ^4^ Center for Plant Science Innovation University of Nebraska‐Lincoln Lincoln Nebraska USA; ^5^ Department of Plant Biology University of Illinois at Urbana‐Champaign Urbana Illinois USA; ^6^ Department of Crop Sciences University of Illinois at Urbana‐Champaign Urbana Illinois USA; ^7^ Present address: School of Biological Sciences Illinois State University Normal Illinois USA

**Keywords:** smart canopy, leaf angle, liguleless, photosynthesis, crop productivity, crop water use

## Abstract

Plant architecture influences the microenvironment throughout the canopy layer. Plants with a more erect leaf architecture allow for an increase in planting densities and allow more light to reach lower canopy leaves. This is predicted to increase crop carbon assimilation. Frictional resistance to wind reduces air movement in the lower canopy, resulting in higher humidity. By increasing the proportion of canopy photosynthesis in the more humid lower canopy, gains in the efficiency of water use might be expected, although this may be slightly offset by the more open erectophile form canopy. An anatomical feature in members of the Poaceae family that impacts leaf angle is the articulated junction of the sheath and blade, which also bares the ligule and auricles. Mutants, which lack ligules and auricles, show no articulation at this junction, resulting in leaves that are near vertical. In maize, these phenotypes termed liguleless result from null mutations of genes: *ZmLG1* (Zm00001eb67740) and *ZmLG2* (Zm00001eb147220). In sorghum, SbiRTx430.06G264300 (*Sb*LG1) and SbiRTx430.03G392300 (*Sb*LG2) are annotated as the respective maize homologues. A hair‐pin element designed to down‐regulate both *Sb*LG1 and *Sb*LG2 was introduced into the grain sorghum genotype RTx430. Derived transgenic events harbouring the hair‐pin failed to develop ligules and displayed reduced leaf angles to the vertical, but less vertical than in null mutations. Under field settings, plots sown with these sorghum events having an erect architecture phenotype displayed an increase in photosynthesis in lower canopy levels, which led to increases in above‐ground biomass and seed yield, without an increase in water use.

## Introduction

Sorghum (*Sorghum bicolor* L. Moench) is a C4 photosynthetic crop and the fifth most important cereal globally in terms of total production (UN‐FAO, 2024). It contributes to the bioeconomy through products that are utilized in food, feed and various industrial applications and is particularly valued for its drought tolerance and as a resilient crop of the semi‐arid tropics (Kanbar *et al*., 2021). It is also a leading target species for the production of biomass as a feedstock for fuel and bioproducts (Mullet *et al*., 2014). Sorghum breeding programmes are continuously seeking genetic variation that can contribute to phenotypic gains in yield, protection of yield and quality of the harvest. Most approaches to boost crop biomass production and yield also increase water use, thus enhancing the risk of a drought stress event occurring (Lobell *et al*., 2014; Ort and Long, 2014; Leakey *et al*., 2019). While irrigation averts drought stress, it accounts for 71% of fresh water use at levels of extraction that are unsustainable (Long, 2025). Therefore, identifying strategies to increase productivity in crops without additional water use is a high priority.

Modern genotypes of most crop plants form canopies of leaves in which the uppermost leaves capture the majority of light; thus, a large percentage of carbon uptake and water loss occurs from these leaves. In full sunlight these leaves are absorbing more light than they can use in photosynthesis, while photosynthesis is strongly light limited in the deeply shaded lower canopy leaves. Biophysical theory predicts that photosynthetic carbon gain, biomass production and water use efficiency (WUE) will be improved by engineering optimal leaf angle distributions to enhance light penetration through the crop canopy, thereby improving the distribution of photosynthetic activity through the canopy profile (Drewry *et al*., 2014; Truong *et al*., 2015). Additionally, wind speed is greatest at the top of the canopy, decreasing progressively due to frictional drag towards the soil surface, which largely uncouples the air in the lower canopy from the bulk atmosphere, allowing humidity to build. Water loss at a given stomatal conductance is proportional to the humidity gradient between the inside of the leaf and the air. So, more photosynthetic activity lower in the canopy where humidity is higher would be associated with less transpiration, allowing a higher WUE. In addition, more light in the lower canopy reduces the loss of photosynthetic efficiency that occurs in sorghum and related species (Collison *et al*., 2020; Jaikumar *et al*., 2021; Liu *et al*., 2024; Pignon *et al*., 2017).

Studies of natural genetic variation in leaf angle have supported this hypothesis and highlighted synergies between more erect leaves and greater planting densities (Cao *et al*., 2022; Jaikumar *et al*., 2021; Mantilla‐Perez *et al*., 2020; Mantilla‐Perez and Fernandez, 2017). Maize liguleless mutant hybrids allow for increases in planting densities and greater yields (Lambert and Johnson, 1978), outcomes also observed in maize lines carrying edits in the B3‐domain transcription factor allele that is associated with erect growth patterns (Tian *et al*., 2019). However, it was not reported whether these yield gains came at the cost of greater water use. This study was designed to test the hypothesis that altering the architecture of a sorghum plant by downregulating the liguleless alleles in the crop would lead to improved yield and biomass production without increasing demand for water.

However, engineering a more erect canopy could lead to closer coupling of the lower canopy air with the bulk atmosphere. This would lower humidity or possibly eliminate any WUE gain from increasing the proportion of canopy photosynthesis that occurs in the lower canopy. This would be offset or nullified if development in the more humid lower canopy results in decreased instantaneous leaf level water use efficiency (*iWUE*), impacting the ratio of net leaf CO_2_ uptake to stomatal conductance (*A*/*g*_s_).

The articulated junction of the leaf sheath and blade, that is the laminar joint which bears the ligule, determines the angle of the blade, often referred to as leaf angle (LA) during development (Tian *et al*., 2019). The ligule is an adaxial membranous appendage connected to the triangle shaped auricle at the junction about sheath and leaf blade (Neher *et al*., 2023). A phenotype was identified in maize germplasm which lacked the laminar joint, auricles and ligule, resulting in much smaller LA to the vertical that is more erect leaves (Emerson, 1912). This phenotype is commonly termed ‘liguleless’. The gene model in maize that primarily governs the liguleless phenotype, *liguleless*‐1 (Zm00001eb67740), is a member of the Squamosa Promoter‐Binding like family of transcription factors (Moreno *et al*., 1997). Members of this gene family are associated with a multitude of plant physiological and developmental processes (Guo *et al*., 2022; Peng *et al*., 2019; Qin *et al*., 2023; Zhou *et al*., 2024). Genotypes homozygous for the recessive *Zmlg*1 allele in maize are characterized by absence of a ligule and blade articulation, coupled with defective development of auricle structures, creating a lack of a blade/sheath boundary, and reduced LA (Becraft *et al*., 1990; Sylvester *et al*., 1990). A second gene model in maize that impacts development of the ligule is liguleless‐2 (*Zm*LG2‐Zm00001eb147220). The recessive *Zmlg*2 mutant has been linked with a developmental stage‐dependent liguleless phenotype, albeit a less severe phenotype relative to maize genotypes homozygous for *Zmlg*1, with observed reduction in auricle formation (Harper and Freeling, 1996; Walsh *et al*., 1998). *Zm*LG2 encodes for a basic‐leucine zipper (bZIP) transcription factor also involved in the establishment of the blade‐sheath boundary (Walsh *et al*., 1998).

Previous research employed mutational approaches to create nulls in liguleless alleles that result in complete loss of the lamina joint and translate to a very small leaf angle of <6° to the vertical phenotype (Brant *et al*., 2021; Lee *et al*., 2007). This strong erect phenotype was proposed as a visual marker for transformation rather than a genetic variant for crop improvement *per se* (Brant *et al*., 2021). The small leaf angle results in blades close to the stem, likely impacting light capture. To achieve a less extreme decrease in leaf angle, a knock‐down rather than knock‐out expression strategy was pursued here. Based on sequence similarity to the translation products of *Zm*LG1 and *Zm*LG2, SbiRTx430.06G264300 and SbiRTx430.03G392300 are the *Sb*LG1 and *Sb*LG2 homologues, with 80% and 93% identity between the respective *Sb*LG1/2 and *Zm*1/2 homologues (Goodstein *et al*., 2012). From this information, a hair‐pin element was designed to constitutively down‐regulate both *Sb*LG1 and *Sb*LG2. The hair‐pin element was subsequently assembled into a binary vector and introduced into sorghum, grain genotype RTx430, to create a sorghum with reduced leaf angles. Derived sorghum events carrying this hair‐pin were characterized at the molecular and phenotypic level under the greenhouse. Three lead independent events selected from the greenhouse phenotyping for erect leaf angle were used for replicated field trials. These sorghum events allowed for the testing of the hypothesis that within the same genetic background, downregulation of *Sb*LG1 and SbLG2 will significantly increase canopy light penetration, photosynthesis, biomass production, and grain yield without more water use.

## Results

### Creation of sorghum events with reduced leaf angle

Primary events carrying the T‐DNA element of the binary vector pPTN1355 (Figure S1) were established in the greenhouse and visually assessed for a vertical leaf blade phenotype. The three selected independent events displaying the phenotype, ZG629‐6‐3a (event 1), ZG630‐5‐27d (event 2) and NN567‐3‐2‐1 (event 3) were further characterized.

**Figure 1:**
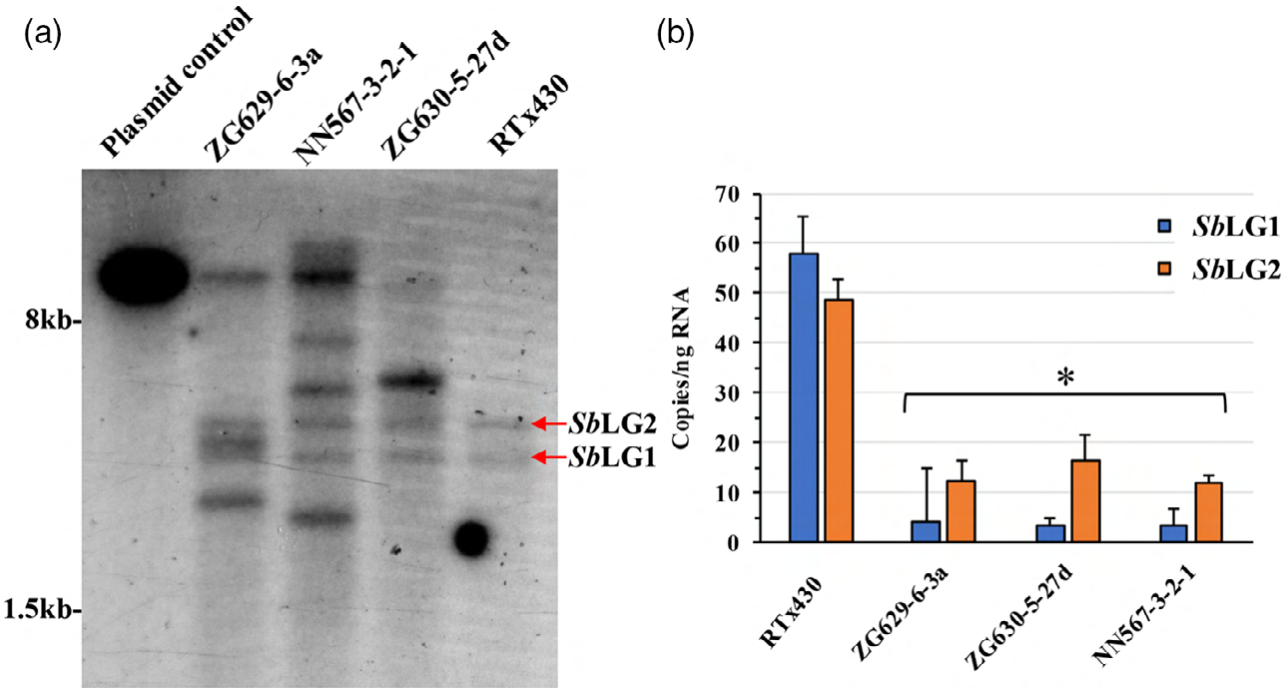
Southern blot analysis and expression profiling of *Sb*LG1 and *Sb*LG2 genes in sorghum events carrying hair‐pin transgenic allele. (a) Southern blot analysis on the *Sb*LG1/*Sb*LG2 hair‐pin events. Lane 1: linearized (digested with *Eco*RV) binary vector pPTN1335. Lanes 2–4: *Eco*RV restriction digest of total genomic DNA from the respective *Sb*LG1/*Sb*LG2 hair‐pin events. Lane 5: *Eco*RV restriction digest of total genomic DNA from wild type control RTx430. Membrane hybridized with the *Sb*LG1/*Sb*LG2 hair‐pin arm labelled [α‐^32^P]‐dCTP probe. The endogenous *Sb*LG1 and *Sb*LG2 signals (red arrows). (b) Transcript profiling of *Sb*LG1 and *Sb*LG2 alleles via droplet digital PCR (ddPCR) assays from the respective hair‐pin events and wild type control RTx430 under greenhouse conditions. Data reported as transcript copies per ng RNA. * indicates significance from wild type.

A Southern blot analysis was conducted on T_2_ population individuals to assess the complexity of the integration of the hair‐pin containing transgenic allele in the genome across the three events (Figure 1). Here genomic DNA was restriction digested with enzyme *Eco*RV. This restriction enzyme was used given that the *Eco*RV site within the T‐DNA element sits in the 35S CaMV promoter (Figure S1) of the selectable marker cassette, which resides proximal to the left border, a hybridization signal larger than 5.2 kb will be suggestive of an intact functional hair‐pin cassette. As can be gleaned from the Southern blot hybridization signals the two bands observed in the wildtype RTx430 lane represent the endogenous *Sb*LG1 and *Sb*LG2 genes. For these loci, an *Eco*RV restriction digested RTx430 genome, hybridization bands are expected to be observed at approximately 3.6 kb and 4.3 kb for the *Sb*LG1 and *Sb*LG2 loci, respectively, which the Southern results reflect (Figure 1). The lack of any additional bands observed in the WT control lane suggests that the hair‐pin design was likely specific to the two liguleless genes. In regard to the event lanes, event ZG629‐6‐3a has three transgenic loci, with one locus with an intact hair‐pin cassette. Event NN567‐3‐2‐1 harbours four transgenic loci, with three functional hair‐pin cassettes, while event ZG630‐5‐27d carries a single functional hair‐pin transgenic locus (Figure 1).

The ability of the intact functional hair‐pin cassette to effectively down‐regulate expression of *Sb*LG1 and *Sb*LG2 alleles was assessed via ddPCR. The assay was carried out on tissue samples around the ligule region from T_3_ individuals from each of the respective events, along with a wildtype, under greenhouse conditions (Figure 1b). The results reflect significant down‐regulation of expression from both genes in tissue sampled from the ligule region, with *Sb*LG1 displaying a reduction of almost 90%, and *Sb*LG2 expression reduced by approximately 30% (Figure 1b). Expression in leaf blade proper tissue was also assessed via both RT‐PCR and ddPCR (Figure S2a,b). Similar reductions in expression of both *Sb*LG1 and *Sb*LG2 were observed, although expression of the two liguleless genes was significantly lower in the leaf blade proper region (Figure S2b), relative to the ligule region (Figure 1b). No change in expression was detected in the reference gene used in the assays, *Sb*EIF4*α*, in tissue samples from both the leaf blade proper and ligule region (Figure S2c).

### Plant architecture changes observed in the sorghum events

**Figure 2:**
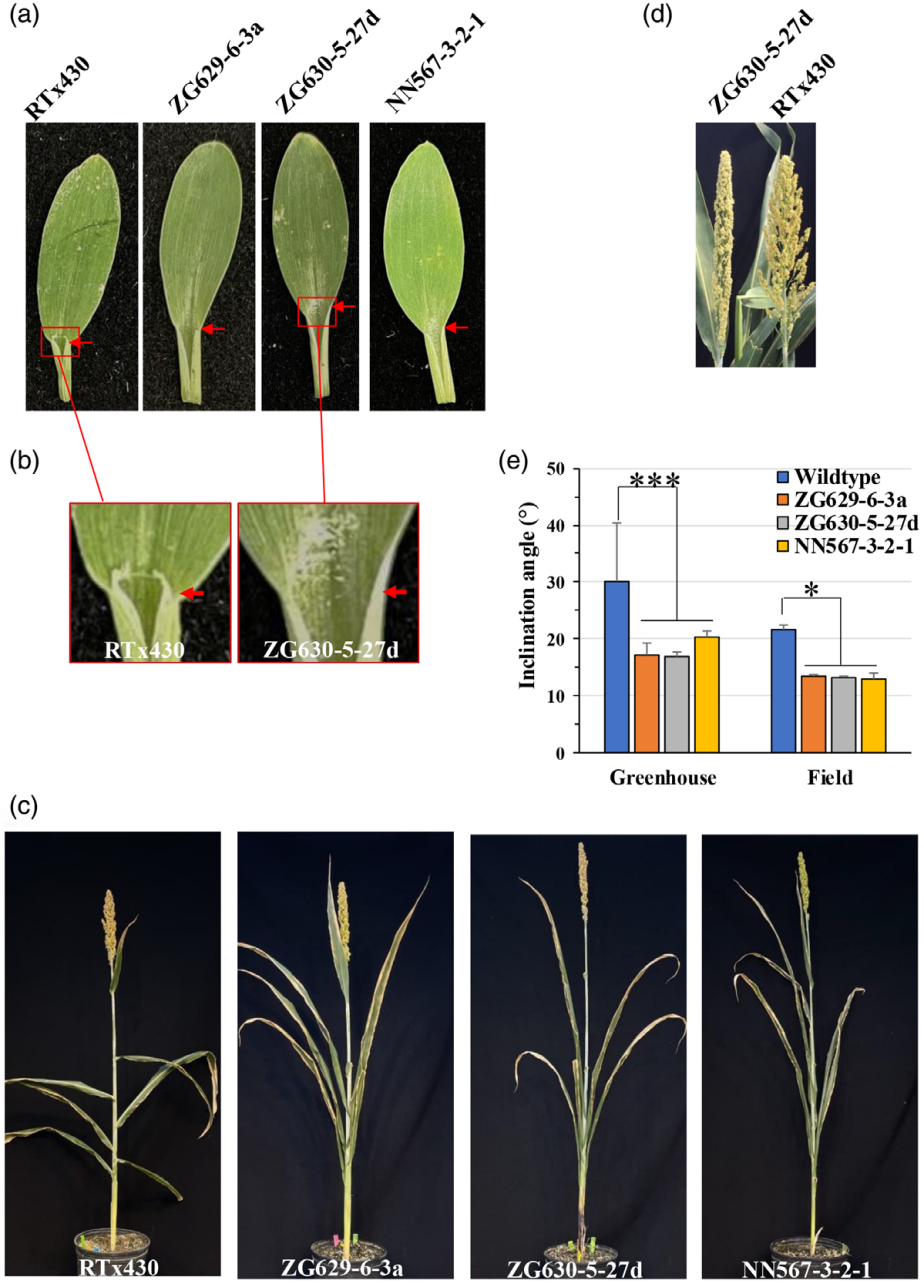
Phenotype of *Sb*LG1/*Sb*LG2 hair‐pin events. (a) Young leaves of control (RTx430) and respective *Sb*LG1/*Sb*LG2 hair‐pin events highlighting the absence of ligule/auricle about the junction of the leaf/sheath in the transgenic events. (b) Close‐up image of the ligule/auricle (red arrow) in wildtype (RTx430) and event ZG630‐5‐27d. (c) Wildtype (RTx430) and representative T_2_ generation individuals from the respective *Sb*LG1/*Sb*LG2 hair‐pin events under greenhouse environment. (d) Close‐up of panicle on a wildtype (RTx430) plant and a T_2_ individual derived from event ZG630‐5‐27d under greenhouse conditions. (e) Assessment of leaf angle (°) changes in wildtype (RTx430) versus respective *Sb*LG1/*Sb*LG2 events under greenhouse and field settings. * (*t*‐test *p* < 0.05) *** (*t*‐test *p* < 0.01).

Phenotypic changes in the presence/absence of a ligule and impact on leaf angle were assessed in the selected events by growing the events and controls side‐by‐side in a greenhouse setting. Developing leaves from individuals derived from the respective events lacked a ligule and boundary at the blade/sheath interface (Figure 2a,b). An upright plant architecture was observed throughout development (Figure 2c), with narrower panicles than in the wildtype (Figure 2d). In the greenhouse, the average angle in the six leaves below the flag leaf was reduced by approximately 30%–40% across the three transgenic events relative to wildtype, with minimal variation from the more apical leaf to the base. However, in the wildtype, leaf angles were larger at the top of the plant relative to those below, hence the larger observed variation in the controls (Figure 2e).

Two field trials were conducted in Illinois (USA) in 2018 and 2020, to assess whether the variation in leaf angle observed under greenhouse settings is maintained under a field environment, and to assess the impact that the architectural changes imposed by down‐regulating *Sb*LG1 and *Sb*LG2 have on various agronomic and physiological traits. The segregation ratios, based on the nptII ELISA screening observed, were event ZG630‐5‐27d (100:0), event NN5673‐2‐1 (98:2) and event ZG629‐6‐3a (97:3). This outcome suggests that in the latter two events, inheritance is closer to a 15:1 ratio. A 94:6 segregation ratio would result if one of the alleles observed in the S. blot is segregating independently from the others. Nonetheless, uniformity of erect architecture phenotype of the field plots sown in 2018 and 2020 with the transgenic events was observed.

Monitoring of leaf angle changes revealed the phenotype of more erect leaf blades is indeed maintained under a field setting (Figure 2e). However, while the leaf angles in the transgenic events were significantly reduced relative to wildtype, the degree of changes was not as large as that observed in the greenhouse, possibly due to the closer spacing in the field than the potted greenhouse plants (Figure 2e).

**Figure 3:**
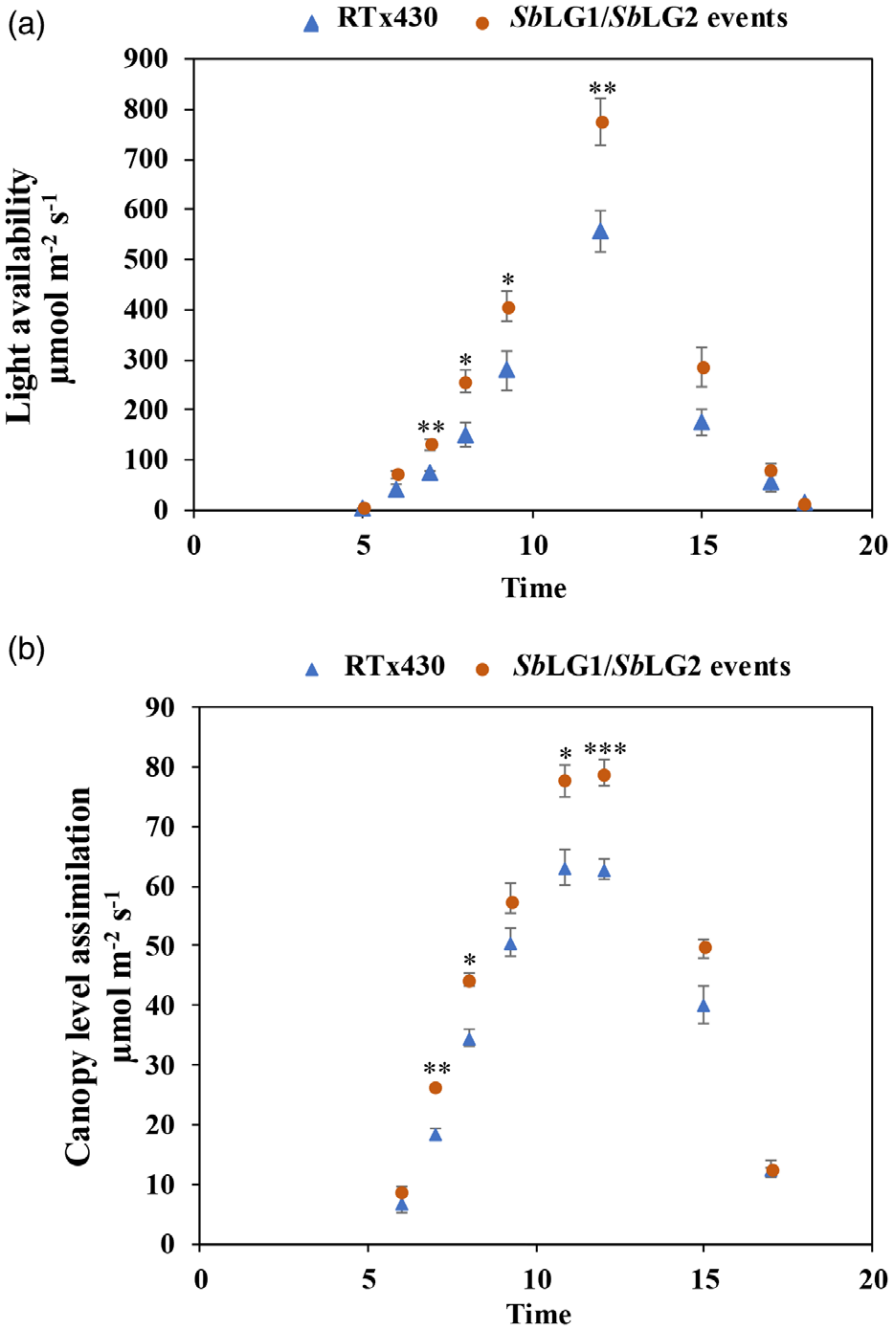
Light availability and instantaneous photosynthetic carbon accumulation (*Acanopy*). (a) Light availability through the canopy measured at 60% of the distance from the top of the canopy to ground level, as a function of time. Data collected from 2020 field plots mid‐August full sun day. (b) Projected instantaneous photosynthetic carbon accumulation, through the canopy, as a function of time. Data on *Sb*LG1/*Sb*LG2 events are means from the three hair‐pin events. Symbols ‘*’, ‘**’, ‘***’ and ‘****’ represent statistically significant differences between the pooled transgenic plants and the wildtype at *α* = 0.05, 0.01, 0.001 and 0.0001, respectively, when these are compared at each time point using an unpaired, unequal variance *t*‐test.

Penetration of PPFD through the canopy was monitored at a depth of 60% from the top of the canopy as a function of time during the day (Figure 3a). Diffuse radiation in the early morning (before 7:00) and late afternoon (after 15:00) reached this position in the lower canopy equally in wildtype versus *Sb*LG1/*Sb*LG2 events. However, as PPFD increased at mid‐day, the proportion of direct to indirect radiation increased, and the amount of PPFD penetrating into the lower canopy became significantly greater in *Sb*LG1/*Sb*LG2 events than wildtype, by approximately 20% at mid‐day (Figure 3a; Table S2). Between 7:00 and 17:00, light extinction coefficients were on average lower across the three transgenic events compared to wildtype (*F*_3,15_ = 6.09, *p* = 0.0064). Consistent with these observations, leaf angle and light extinction coefficient values (*k*_ext_) assessed at both late vegetative and at booting stages were significantly reduced across the transgenic events, relative to wildtype plots (Tables 1 and 2).

**Table 1:** Light extinction coefficient (*K*_ext_) and leaf angle measurements (field 2018 and 2020)

Event	*K*_ext,midday_	Leaf angle^†^	*K*_ext,midday_^†^	*K*_ext,midday_	Leaf angle^†^
Stage	Boot	Vegetative	Vegetative	Boot	Vegetative
Year	2018	2018	2020	2020	2020
	–	–	–	–	–
Wildtype	2.94 ± 0.14	21.7 ± 0.8	1.86 ± 0.26	2.68 ± 0.09	22.5 ± 0.4
NN567‐3‐2‐1	1.71 ± 0.21*	13.0 ± 0.2***	1.28 ± 0.14	1.88 ± 0.09***	12.7 ± 0.2***
ZG629‐6‐3a	1.72 ± 0.28*	13.4 ± 0.4***	1.23 ± 0.12	1.77 ± 0.19***	12.3 ± 0.3***
ZG630‐5‐27d	1.84 ± 0.19*	13.3 ± 0.3***	1.14 ± 0.08*	2.00 ± 0.10**	12.4 ± 0.4***

Variable	Analysis of variance				
			*F*_8,15_	*F*_8,15_	*F*_8,15_
Event	6.52*	–	–	12.55***	–

Variable	Kruskal–Wallis				
	*χ* ^2^	*χ* ^2^	*χ* ^2^	*χ* ^2^	*χ* ^2^
Event	–	8.97*	5.79	–	13.425**

Leaf inclination angle and light extinction coefficient (*k*_ext_) taken on transgenic events during 2018 and 2020 under a field environment. Event column refers to the respective events and to the control, RTx430. Extinction coefficients were measured through the canopy at noon on a sunny day, during boot stage in 2018 and during both boot and late vegetative stages in 2020. Leaf inclination angle was tabulated from the youngest fully expanded leaf at late vegetative stage. Data presented are means (± standard error) and results of statistical significance tests (including either analysis of variance or Kruskal–Wallis testing, depending on whether data were normally distributed). Symbols ‘*’, ‘**’, ‘***’ and ‘****’ represent statistical significance at *α* = 0.05, 0.01, 0.001 and 0.0001, respectively. For individual cell means, such symbols refer to statistical significance when comparisons are made against the wildtype. Symbol ‘^†^’ indicates anon normally distributed variable, as assessed by the Shapiro–Wilk test. Sample size was *n* = 6 in 2020 and *n* = 4 in 2018.

**Table 2:** *A*_canopy_ and yield parameters (field 2018)

Event	*A*_canopy,midday_	Veg. biomass	Grain yield	H.I.
Stage	Boot, μmol/m^2^/s	Harvest, Mg/ha	Harvest, Mg/ha	Harvest, %
Wildtype	62.21 ± 2.62	4.49 ± 0.37	2.88 ± 0.19	32.3 ± 1.5
NN567‐3‐2‐1	89.84 ± 5.73*	5.65 ± 0.16***	3.10 ± 0.16*	27.4 ± 0.9***
ZG629‐6‐3a	90.21 ± 7.12*	5.70 ± 0.34***	3.07 ± 0.19	26.2 ± 0.6***
ZG630‐5‐27d	86.42 ± 5.02	5.73 ± 0.22***	3.05 ± 0.15	26.6 ± 1.1***

Variable	Analysis of variance			
	*F*_6,9_	*F*_6,9_	*F*_6,9_	*F*_6,9_
Event	5.06*	22.00***	4.50*	21.96***

Estimated instantaneous carbon assimilation at midday through the length of the canopy (*A*_canopy,midday_), vegetative biomass yield, grain yield and H.I. (harvest index) in wildtype RTx430 and three transgenic sorghum events (*n* = 4). Canopy carbon assimilation was estimated at boot stage while other variables were measured at harvest. Estimated carbon assimilation values were inferred from photosynthetic light response curves, combined with light measurements through the canopy. Data presented are means (± standard error) and analysis of variance results. For *F*‐values, symbols ‘*’, ‘**’ ‘***’ and ‘****’ represent statistical significance at *α* = 0.05, 0.01, 0.001 and 0.0001, respectively. For individual cell means, such symbols refer to statistical significance when comparisons are made against the wildtype. Data are from a 2018 field experiment in Savoy, IL. Sample size was *n* = 4.

Projected whole canopy carbon assimilation (*A*_canopy_) in the transgenics was increased throughout the day mirroring the improved light penetration (Figure 3b). Significant increases in mid‐day *A*_canopy_ of the transgenic events relative to wildtype at mid‐day were found in both years (Tables 2 and 3). In 2020 this difference was present at boot stage (*α* = 0.05) by ANOVA test, but not at late vegetative stage, possibly because of greater canopy closure and more intense self‐shading at boot stage (Table 3). Two of the transgenic events in 2018, and all three when measured at boot stage in 2020, had significantly higher midday *A*_canopy_ than wildtype (Tables 2 and 3). When trends from both years were combined using Fisher's combined test, all three events showed significantly higher *A*_canopy_ than wildtype (*p* = 0.0014, *p* = 0.0005 and *p* = 0.0052 for events NN567‐3‐2‐1, ZG629‐6‐3a and ZG630‐5‐27d, respectively, combining vegetative stage and boot stage data for 2020 and combining *p*‐values from across both years). At boot stage, increased whole canopy assimilation over the course of the day amounts to an additional 28% or 20 g/m^2^/day in the transgenic plots compared to controls (Table 3).

**Table 3:** *A*_canopy_ and yield parameters (field 2020)

Event	*A*_canopy,midday_^†^	*A*_canopy,midday_	*A*_cumulative_	Veg. biomass	Grain yield	H.I.
Stage	Vegetative, μmol/m^2^/s	Boot, μmol/m^2^/s	Boot, g/m^2^/day	Harvest, Mg/ha	Harvest, Mg/ha	Harvest, %
Wildtype	96.32 ± 6.15	62.84 ± 1.61	65.64 ± 1.51	5.39 ± 0.20	2.49 ± 0.10	31.6 ± 0.7
NN567‐3‐2‐1	109.98 ± 3.32	78.93 + 1.94**	84.16 ± 3.91**	6.66 ± 0.28***	2.62 ± 0.08*	28.4 ± 0.9***
ZG629‐6‐3a	111.05 ± 2.91	81.73 ± 4.31***	86.61 ± 4.32***	6.41 ± 0.16***	2.61 ± 0.11*	28.8 ± 0.7***
ZG630‐5‐27d	113.27 ± 1.83*	76.47 ± 2.26**	81.88 ± 2.26**	6.54 ± 0.20***	2.59 ± 0.10	28.3 ± 0.7***

Variable	Analysis of variance					
	*F*_8,15_	*F*_8,15_	*F*_8,15_	*F*_8,15_	*F*_8,15_	*F*_8,15_
Event	3.37*	11.27***	7.09**	24.08****	4.96*	21.51***

Estimated instantaneous carbon assimilation at midday through the length of the canopy (*A*_canopy,midday_), cumulative carbon accumulation through the canopy over the course of the day (*A*_cumulative_), vegetative biomass yield, grain yield and harvest index in wildtype RTx430 and three transgenic sorghum events (*n* = 6). Values for *A*_canopy,midday_ were estimated both at late vegetative and at boot stage; *A*_cumulative_ was estimated only during boot stage, while grain and biomass yield were estimated at harvest. Data on estimated carbon assimilation were inferred from photosynthetic light response curves, measured both at late vegetative stage and at boot stage, combined with light measurements through the canopy. Data presented are means (± standard error) and results of analysis of variance testing. *F*‐values, symbols ‘*’, ‘**’, ‘***’ and ‘****’ represent statistical significance at *α* = 0.05, 0.01, 0.001 and 0.0001, respectively. For individual cell means, such symbols refer to statistical significance when comparisons are made against the wildtype. Symbol ‘^†^’ indicates a non‐normally distributed variable, as assessed by the Shapiro–Wilk test. Data are from a 2020 field experiment in Savoy, IL. Sample size was *n* = 6.

**Figure 4:**
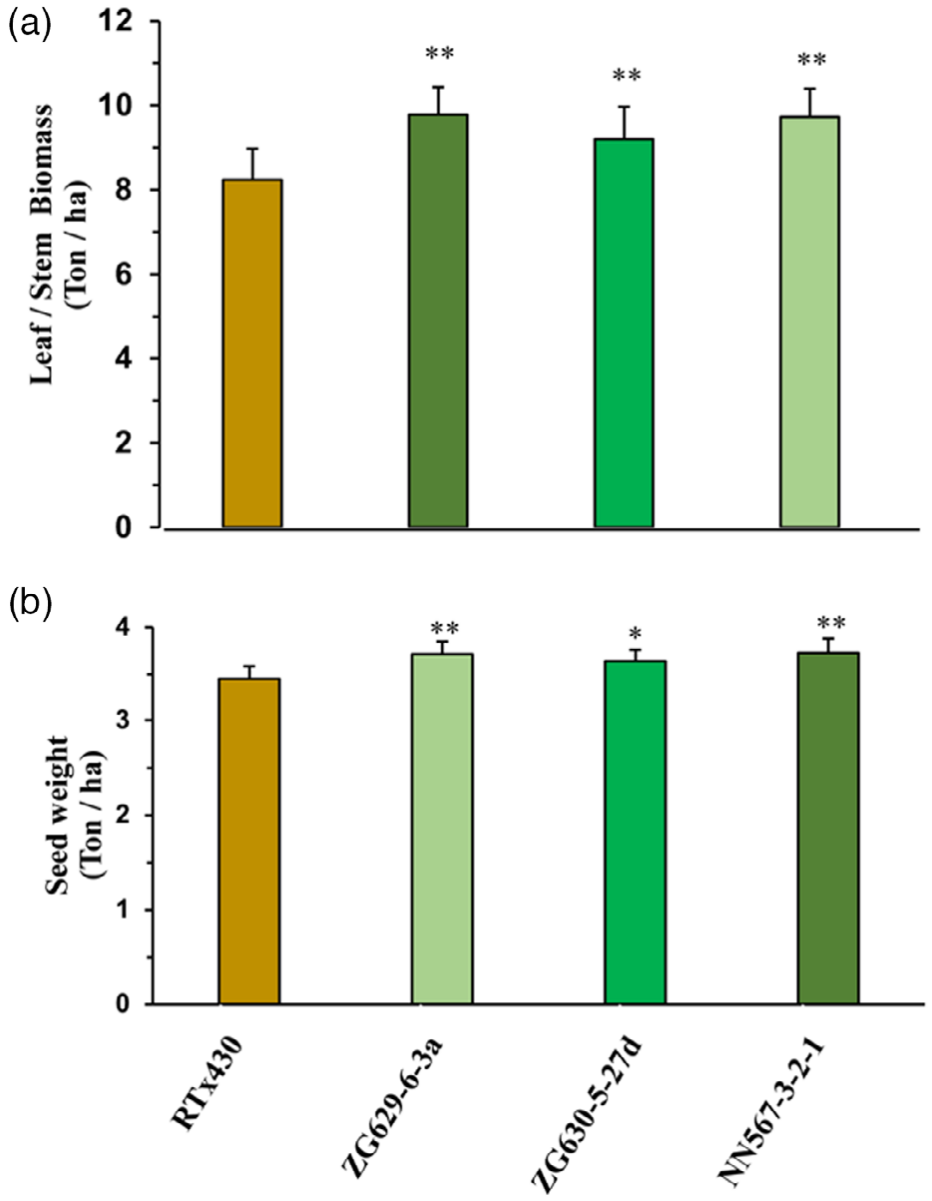
Sorghum biomass and yield estimates from field plots. (a) Dry biomass (leaf and stem) on a ton per hectare calculation obtained from 2020 field plots on wildtype (RTx430) and respective *Sb*LG1/*Sb*LG2 hair‐pin events. (b) Seed weights on a ton per hectare calculation obtained from 2020 field plots on wildtype (RTx430) and respective *Sb*LG1/*Sb*LG2 hair‐pin events. * and ** indicate significance at *α* = 0.05 and *α* = 0.01, respectively.

In both 2018 and 2020, all three transgenic events significantly outperformed the wildtype RTx430 in terms of above‐ground biomass (ca. +24%) and grain yield (ca. +5.5%) (Figure 4; Tables 2 and 3). Vegetative biomass was significantly higher in each individual event compared to wildtype in both years. Due to the small effect sizes, grain yield for only one of the individual events in 2018 and two of the three events in 2020 was significantly higher than wildtype after individual pairwise comparison. However, when the observed trends in grain yield were combined across both years using Fisher's combined test, each event showed a significantly higher yield than wildtype (*p* = 0.0003, *p* = 0.01012 and *p* = 0.0067 for events NN567‐3‐2‐1, ZG629‐6‐3a and ZG630‐5‐27d, respectively) (Figure 4b). The significant increases in grain yield were achieved despite a significant decrease (ca. −14%) in harvest index in all three transgenic events in both years (Tables 2 and 3).

Across both years, upper canopy leaves had greater light‐saturated photosynthetic rates (*A*_*s*at_), greater electron transport rates (*J*_max_), higher apparent quantum yields of assimilation under light‐limiting conditions (*Φ*_CO2,app,max_), higher light‐adapted respiration rates (*R*_D_) and a reduced ratio of light‐limited electron transport rate to assimilation rate (*Φ*_J_/*Φ*_CO2,max,app_) than lower canopy leaves. These results confirm previous observations in biomass sorghum genotypes (Jaikumar *et al*., 2021) and in maize and miscanthus (Pignon *et al*., 2017). However, events did not differ with respect to any of the leaf‐level parameters, nor was there an interaction effect between canopy level and event, indicating that the altered leaf angle phenotype did not modulate the effects of self‐shading on leaf‐level parameters. This suggests that the improvements in cumulative carbon assimilation, biomass yield and grain yield in the transgenic events are due to direct alteration of the light environment rather than second‐order effects on leaf‐level photosynthetic parameters. Several of these parameters, in 2020, showed a stage by level interaction, indicating that the effects of shading on leaf physiology might become more severe later in the season as the shade environment is more intense. Data on carbon assimilation parameters, extracted from the light response curves of leaf CO_2_ uptake, are shown in Tables S3 and S4 for 2018 and 2020, respectively: data on electron transport parameters are shown in Tables S5 and S6 for 2018 and 2020, respectively.

**Figure 5:**
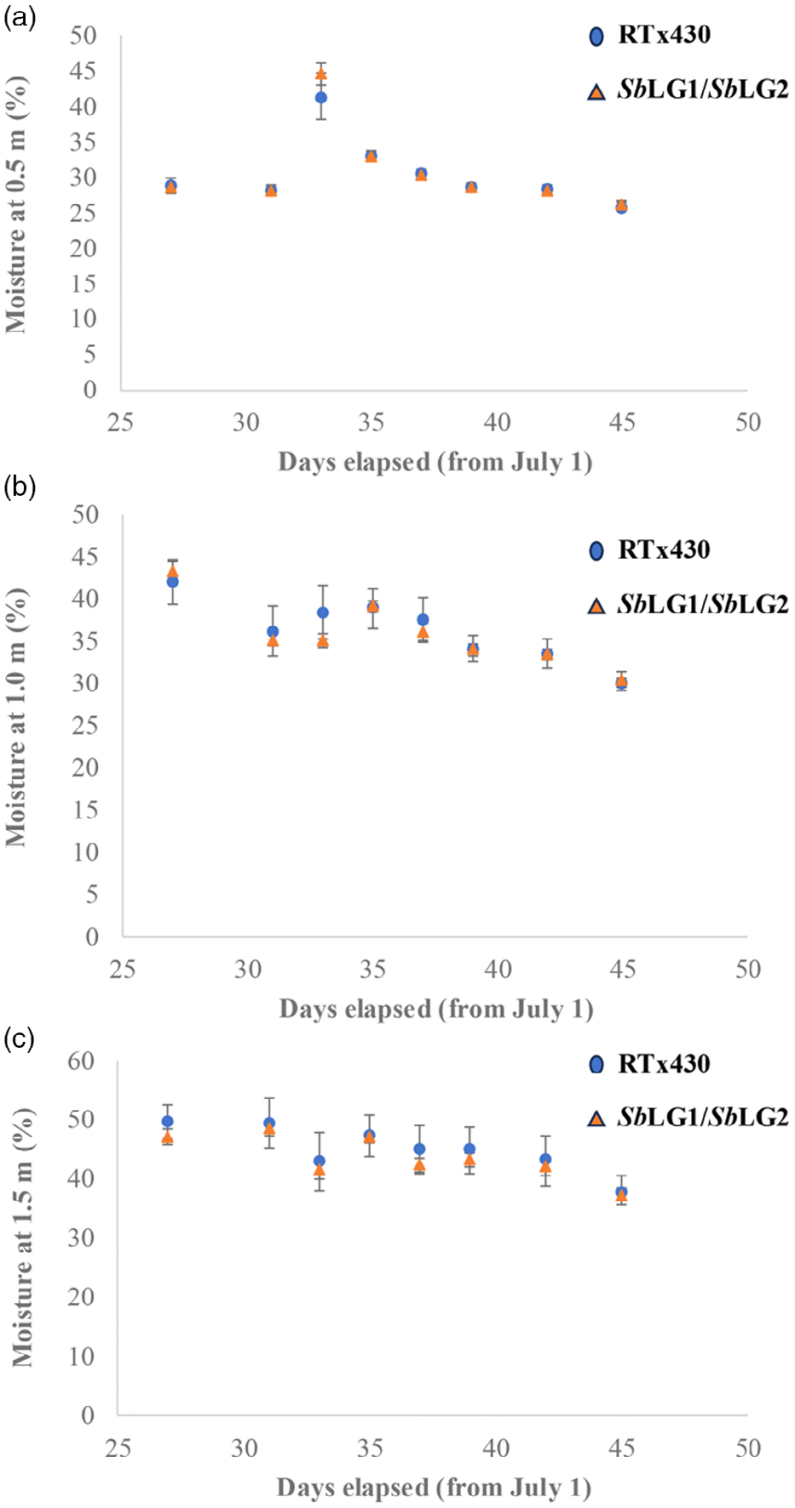
Soil moisture within plots of wildtype (RTx430) and SbLG1/SbLG2 hairpin events. (a) Soil moisture at 0.5 m depth, within plots (*n* = 6) of wild type sorghum (RTx430) and transgenic *Sb*LG1/*Sb*LG2 hair‐pin events. The transgenic values are averaged among three separate transgenic events. Data are from a 2020 field experiment. (b) Soil moisture at 1.0 m depth, within plots (*n* = 6) of wild type sorghum (RTx430) and transgenic *Sb*LG1/*Sb*LG2 hair‐pin events. The transgenic values are averaged among three separate transgenic events. Data are from a 2020 field experiment. (c) Soil moisture at 1.5 m depth, within plots (*n* = 6) of wild type sorghum (RTx430) and transgenic *Sb*LG1/*Sb*LG2 hair‐pin events. The transgenic values are averaged among three separate transgenic events. Data are from a 2020 field experiment.

These increases in crop photosynthesis, above‐ground biomass and yield were achieved without any increase in crop water use, as the plots sown with the transgenic events did not show a significant decrease in soil moisture levels at either 0.5, 1.0 or 1.5 m depth (Figure 5) despite higher photosynthesis and productivity. Analysis of variance results in both 2018 and 2020 showed no significant effect of ‘event’ or of the event by date interaction, while no events showed consistently higher or lower moisture levels than wildtype, as assessed via individual *post hoc* comparisons at each date (Tables S7 and S8). Average soil moisture over the sampling period was also not different in any of the events compared to wildtype, either within individual years or when combined across both years by Fisher's combined test.

Leaf‐level *iWUE* (*A*/*g*_s_) measured under the same constant conditions, including a fixed humidity, was not affected by event or interactions with depth into the canopy or with growth stage (Table S9). This shows that there was no acclimatory loss of leaf‐level WUE with either the transgenic events or depth into the canopy. Leaf‐level transpiration is the product of *g*_s_ and the humidity gradient from the water vapour saturated leaf intercellular spaces to the air surrounding the leaf. It follows that if *A* is increased in the lower canopy, but *A*/*g*_s_ remains constant, then under the more humid conditions of the lower canopy, the WUE of the lower canopy and in turn crop productivity will be higher.

Canopy level estimates on stomatal conductance and *iWUE* over the course of the day revealed improvements in both parameters in the respective transgenic events, relative to wild type, but with significant increases tending to be detected at boot stage (Table 4).

**Table 4:** Water relation parameters under field environment (2018 and 2020 seasons)

Event	*G*_S,canopy,average_	*IWUE*_canopy,average_	*G*_S,canopy,average_	*IWUE*_canopy,average_	*G*_S,canopy,average_	*IWUE*_canopy,average_
Year	2018	2018	2020	2020	2020	2020
Stage	Boot	Boot	Vegetative	Vegetative	Boot	Boot
Time	Midday	Midday	Midday	Midday	Diurnal	Diurnal^†^
	μmol/m^2^/s	μmol/m^2^/s	mol/m^2^/s	μmol/mol	mol/m^2^/s	μmol/mol
Wildtype	0.096 ± 0.002	161.2 ± 0.1	0.153 ± 0.003	157 ± 11	0.089± 0.001	110 ± 1
NN567‐3‐2‐1	0.120 ± 0.003	187.3 + 1.5 **	0.183 ± 0.003***	151 ± 6	0.110 ± 0.004***	104 ± 1
ZG629‐6‐3a	0.169 ± 0.007	210.1 ± 0.2 ***	0.169 ± 0.007	166 ± 9	0.067 ± 0.003***	187 *±* 10***
ZG630‐5‐27d	0.167 ± 0.004***	147.0 ± 4.0	0.167 ± 0.004	170 ± 4	0.111 ± 0.002***	91 ± 1

	Analysis of variance					
	*F*_6,9_	*F*_6,9_	*F*_8,15_	*F*_8,15_	*F*_8,15_	*F*_8,15_
Event	12.40**	36.81****	9.12**	1.23	54.86****	75.46****

Estimated instantaneous stomatal conductance at midday through the length of the canopy (*G*_S_,_canopy,midday_), average stomatal conductance through the canopy over the course of the day (*G*_S_,_canopy,average_), average intrinsic water use efficiency through the length of the canopy at midday (*IWUE*_canopy_,_midday_) and average intrinsic water use efficiency through the canopy over the course of the day (*IWUE*_canopy,average_), in wildtype RTx430 and three transgenic sorghum events (*n* = 6). All values were estimated at boot stage. Data on estimated stomatal conductance and water use efficiency were inferred from photosynthetic light response curves, measured at boot stage, combined with light measurements through the canopy. Data presented are means (± standard error) and results of analysis of variance testing. For *F*‐values, symbols ‘*’, ‘**’, ‘***’ and ‘****’ represent statistical significance at *α* = 0.05, 0.01, 0.001 and 0.0001, respectively. For individual cell means, such symbols refer to statistical significance when comparisons are made against the wildtype. Symbol ‘†’ indicates a non‐normally distributed variable, as assessed by the Shapiro–Wilk test. Data are from a pair of field experiments (2018 and 2020) in Savoy, IL. Sample size was *n =* 4 in 2018 and *n* = 6 in 2020.

## Discussion

This study successfully addressed its aim of providing proof‐of‐concept for engineering sorghum leaf angle to a lower degree to confer greater photosynthetic carbon gain, biomass production, and yield without greater extraction of soil water (Tables 1, 2, 3, S7 and S8). Sorghum events carrying the dual *Sb*LG1/*Sb*LG2 hair‐pin transgenic allele were significantly down‐regulated in expression of the targeted gene models SbiRTx430.06G256300 (*Sb*LG1) and SbiRTx430.03G392300 (*Sb*LG2). This design was created to change leaf angle in sorghum, given the association with ligule and auricle formation and articulation at the junction of the leaf, known to influence leaf blade angle and plant architecture in maize (Li *et al*., 2015; Moreno *et al*., 1997; Tian *et al*., 2011; Walsh *et al*., 1998) and other monocots (Lee *et al*., 2007; Qin *et al*., 2023; Zhang *et al*., 2025). A genome editing approach can also be employed to create null mutations in *Sb*LG1/*Sb*LG2 alleles; however, this would likely result in near vertical against the stem architecture, which would be detrimental to light interception. This outcome is reflected in the results observed when editing reagents were introduced into sorghum targeting the *Sb*LG1 allele, in which derived homozygous nulls displayed leaf angles of only between 1.5° and 5.4° relative to vertical (Brant *et al*., 2021). However, it would also be possible to achieve the more desirable knock‐down rather than knock‐out by designing editing reagents that target the regulatory regions of the *Sb*LG1 and *Sb*LG2 genes (Tang and Zhang, 2023; Quach *et al*., 2025).

Weather data information for the field sites for 2018 and 2020 (Table S10) shows that 2020 was a drier year, with slightly higher temperatures. At the site, transpiration rates are high and significant soil drying can be reliably observed. As a result, we have used it as a sensitive measure of evapotranspiration on many occasions over a 20‐year period (Gray *et al*., 2016; Hussain *et al*., 2013; Leakey *et al*., 2006; Markelz *et al*., 2011). Soil moisture levels did not differ across the plots, transgenic events and wildtype, consistent with the hypothesized improvement of crop level WUE. However, the variability between plots and time points to soil moisture could have hidden subtle differences. Plot size did not allow for direct measurement of transpiration at the crop level. At the leaf level, the intrinsic WUE under standardized environmental conditions was unchanged with depth into the canopy. This indicates that there is not acclimation of *iWUE* to altered growth light environment. As shown (Tables 2 and 3), since a greater fraction of CO_2_ uptake will occur in the lower canopy and more humidity, crop WUE should rise.

The outcomes of down‐regulating expression of the *Sb*LG1/*Sb*LG2 alleles in sorghum (Figures 1b and S2b) were a reduction in ligule/auricle formation (Figure 2a,b), smaller leaf angles, relative to wildtype (Figure 2e), coupled with changes in panicle architecture (Figure 2d). These are phenotypes previously observed in various monocot species carrying genetic variation that leads to reduced expression of liguleless alleles (Qin *et al*., 2023). The observed leaf angle changes, from an average of 30.2° in parental RTx430, down to 17.2–21.0° in transgenics, were detected under greenhouse settings. Similar outcomes were seen under field conditions, but with an overall leaf angle reduction in both WT and the events, possibly due to higher planting density in the field (Figure 2e).

The sorghum genotype used in this study, RTx430 (Miller, 1984), carries the recessive dwarfing allele *dw*3 (Multani *et al*., 2003), which is a homologue of the maize *Br*2 allele, functionally annotated as an ATP‐binding cassette type B1 auxin efflux transporter. The *dw*3 recessive allele carries an 882 bp duplication in exon 5 of the gene model, rendering it inactive as an auxin transporter. Sorghum genotypes homozygous for *dw*3 display shorter internodes, and in some genetic backgrounds, a reduction in above‐ground biomass and grain yield (George‐Jaeggli *et al*., 2013). In multiple mapping studies, the sorghum *dw*3 allele has been identified as a major genetic determinant of leaf angle (Natukunda *et al*., 2022; Tross *et al*., 2021; Truong *et al*., 2015), which in the BTx623 genetic background (Paterson *et al*., 2009), the dw3/dw3 genotype results in smaller leaf angles (Truong *et al*., 2015). However, while the dwarf genotype RTx430 is homozygous for *dw*3, its leaf angle is relatively large (Figure 2c), which suggests that a nonfunctional *dw*3 allele alone is not sufficient to translate to a reduced leaf angle phenotype, and perhaps the other gene models, identified via genome association type activities (Natukunda *et al*., 2022; Takanashi, 2023; Zhi *et al*., 2022) for this trait are either linked to *dw*3 on chromosome 7, or other non‐linked alleles identified play a role, alone or stacked with *dw*3 in governing plant architecture in sorghum. Interestingly, in the reported association mapping studies, the sorghum liguleless alleles do not pop up as major players in impacting leaf angle in sorghum (Natukunda *et al*., 2022; Tross *et al*., 2021; Truong *et al*., 2015). However, Natukunda *et al*. (2022) did monitor the expression of the sorghum homologues of maize liguleless alleles, including *Sb*LG1/*Sb*LG2. Differential expression analysis of SbiRTx430.06G264300 [Sobic.006G247700] (LG1) *and* SbiRTx430.03G392300 [Sobic.003G363600] (LG2), across canopy layers revealed that *Sb*LG1*/Sb*LG2 are both differentially expressed in tissues about the blade/sheath junction across canopy levels, suggestive of genetic variation within the promoter regions of the alleles.

Screening a diversity panel of 869 photoperiod‐sensitive sorghum (‘biomass’) genotypes (dos Santos *et al*., 2020; Valluru *et al*., 2019), showed a wide variation in leaf angle (Jaikumar *et al*., 2021). Erectophile (more erect leaf angle) genotypes ranged in leaf angle 9–12°, while planophile (more flat leaf angle) genotypes varied in leaf angle 44–48°. The erectophile phenotype was associated with significantly higher above‐ground biomass production in multi‐plot field trials relative to planophile. However, other genetic differences conferring higher biomass yield could have been associated with the genotypes with more erectophile forms. Down‐regulating expression of *Sb*LG1/*Sb*LG2 alleles in an inbred genotype provided the opportunity to test the hypothesis that more vertical leaves allowing better light usage by the canopy would directly result in higher canopy photosynthesis, shoot total biomass and grain yield without increasing water use. This hypothesis was supported in both growing seasons. Using replicated four‐row plots, grain yield, above‐ground biomass, light distribution within the canopy and crop photosynthesis were all significantly increased in three independent sorghum events relative to the wildtype RTx430 (Figure 3; Tables 2 and 3). Harvest index, the proportion of the shoot biomass in grain, was significantly decreased (Tables 2 and 3). Model estimates of daily canopy CO_2_ uptake were increased by 28% at booting, yet final grain yield by 5% (Table 3). These smaller increases in grain yield may reflect the change in panicle form caused by down‐regulation of the liguleless genes (Figure 2e), but might also reflect the fact that RTx430 is a 40‐year‐old inbred (Miller, 1984) that may be sink‐limited, that is have insufficient capacity to utilize all the extra photosynthate in the formation of additional grain. The latter interpretation is consistent with crop harvest index generally being reduced when biomass production is improved through stimulation of photosynthetic carbon gain by elevated [CO_2_] (Bishop *et al*., 2014).

Some of the benefits of a more erect plant architecture in cropping systems are well documented (Cao *et al*., 2022; Pendleton *et al*., 1968; Yan *et al*., 2024; Zhi *et al*., 2022). One such attribute of a more erect canopy is the impact on PPFD, a measurement of photons within the photosynthetic active range (400–700 nm), passing through a monitored area, a physiological parameter that is enhanced in more erect canopies (Long *et al*., 2006; Murchie and Niyogi, 2011). This outcome reflects the improvement in light penetration through the canopy, an effect observed at the field plot level of the *Sb*LG1/*Sb*LG2 hair‐pin events, where elevation in *A*_canopy_, a measure of carbon capture through the canopy, was observed (Figure 3b; Tables 2 and 3). The benefits of improved water use are especially strong when they coincide with times when vapour pressure deficit is the greatest that is midday. The increase in light penetration to the lower canopy in liguleless knockdown plants compared to wildtype was especially strong at midday. This provides a reason to expect, as shown in this work, that photosynthetic carbon gain and productivity can be achieved without increasing crop water use, thus avoiding trade‐offs between productivity and sensitivity to drought (Leakey *et al*., 2019; Lobell *et al*., 2014).

The sorghum liguleless alleles are clearly a target for reducing expression in order to achieve a more erect plant architecture. The challenge, though, is to design a genetic strategy that can create the ‘smart canopy’ (Mantilla‐Perez *et al*., 2020; Ort *et al*., 2015) in sorghum to maximize planting density and carbon capture through the canopy. While the exact architecture to create the ‘smart canopy’ is something that will require a more experimentation to build, a simplistic view of a ‘smart canopy’ would be to have larger leaf angles at the lower portion of the canopy and more upright leaves at the top. The challenge in creating a ‘smart canopy’ via editing of sorghum liguleless alleles, targeting either the gene body *per se* or regulatory regions of the gene models, would be in the designing of editing reagents that can be developmentally regulated. By employing a hair‐pin strategy, one can incorporate a regulatory element upstream of the *Sb*LG1/*Sb*LG2 hair‐pin that is active at 5th leaf/growing point differentiation stages of development through booting, preferably with a promoter active only within the ligule and auricle region of the leaf. Such a design theoretically would manifest a step in the right direction towards a ‘smart canopy’ architecture, with the benefits of mitigating potential off phenotypes (Kolkman *et al*., 2020), due to tissue preferred down‐regulation of *Sb*LG1/*Sb*LG2, and unlike a genome editing approach, the transgenic hair‐pin allele will be dominant, thus a bit more user friendly in sorghum breeding programs.

The findings communicated here are an important step in a design‐build‐test‐learn cycle towards the creation of the ideotype sorghum ‘smart canopy’, showing measured increase in leaf blade angle as a means to sustainably deliver boosts in both grain and biomass for the bioeconomy.

## Materials and methods

### Assembly of binary vector pPTN1335

The hair‐pin arm was synthesized (GenScript Piscataway, NJ USA) to include 250 bp of the 5′ region of the transcript of gene models SbiRTx430.06G264300 and SbiRTx430.03G392300, incorporating *Bam*HI and *Xba*I restriction sites at the 5′ and 3′ ends of the arm, respectively. The hair‐pin arm was subcloned into the vector pUCRNAi‐intron (gift from Heriberto Cerutti, University of Nebraska) which carries the second intron of the Arabidopsis small nuclear riboprotein (At4G02840). The intron is delineated by a series of compatible restriction sites to allow for the hair‐pin arm inverted repeat to be assembled. In this case, *Bam*HI/*Xba*I on one side and *SpeI*/*Bgl*II on the other side of the intron. The derived hair‐pin was subsequently subcloned between the maize ubiquitin1 promoter (Christensen *et al*., 1992) and the cauliflower mosaic virus 35S terminator of transcription. The resultant hair‐pin cassette was subcloned into the binary vector pZP212 (Hajdukiewicz *et al*., 1994), and the final vector designated pPTN1355 (Figure S1).

### Sorghum transformation

The pPTN1355 binary vector was mobilized into *A. tumefaciens* strain NTL_4_/pTiPKPSF_2_ (Luo *et al*., 2001), and the resultant transconjugant was used in sorghum transformation with the grain genotype RTx430 (Miller, 1984) as previously described (Guo *et al*., 2015; Howe *et al*., 2006).

### Molecular characterization of the transgenic events

A total of 13 independent events were generated from the sorghum transformations conducted with the binary vector pPTN1355. Among the primary events, eight displayed an erect phenotype in the greenhouse, from which progeny derived from three of the events were selected for further characterizations. The events were sown in soil‐less potting medium (Metromix 900: SunGro Horticulture, Agawam, MA, USA), fertilized with 80 g slow‐release Osmocote (17% N, 5% P_2_O_5_, 11% K_2_O, 5% S, 2.3% Fe, 0.14% Cu, 0.35% Mn, 0.14% Zn, 0.007% Mb). Plants were grown under supplemental lighting (photosynthetically active radiation of ca. 600 μmol/m^2^/s at daytime peak), under photoperiod from 6:00 to 22:00 h, and temperature maintained between 24 °C (nighttime low) and 31 °C (daytime high). Plants were watered to field capacity on alternate days. Wildtype plants from the stock used for these transformations were grown alongside the transgenics and through the same generations.

Seed increases for the selected independent events were carried out for three generations under a greenhouse environment at the University of Illinois, for use in field trials. Plants were allowed to self‐pollinate, with 6–8 plants per cycle used to increase seed. Each generation was screened using an nptII ELISA kit following the manufacturer's instructions (Agdia, Inc Elkhart, IN, USA). At T_3_ generation 100 plants per event were monitored to assess zygosity of the transgenic allele.

The complexity of the transgenic allele present in the selected lead transgenic events was assessed via Southern blot analysis as previously described (Kumar *et al*., 2012). Isolated total genomic DNA was digested with the restriction enzyme *Eco*RV and the digested DNA separated on a 1% agarose gel and subsequently transferred to a nylon membrane. The membrane was hybridized with a [α‐^32^P]‐dCTP labelled 390 bp element isolated from the *Sb*LG1/*Sb*LG2 hair‐pin arm.

RT‐PCR and droplet digital PCR (ddPCR) assays were conducted on seedlings at the four‐leaf stage. Tissue samples were collected from the youngest fully expanded leaf, about the ligule region, approximately 4 mm area about the sheath/blade junction, and leaf blade proper, that is middle area of the blade. RNA preparations were carried out using RNeasy Plant Mini Kit (Qiagen Inc, Germantown, MD, USA Cat#74904) following the manufacturer's protocol. Synthesis of cDNA was carried out using High Capacity cDNA Reverse Transcription Kit (ThermoFisher Scientific, USA Cat#4368814). For RT‐PCR (CFX96 system, Bio‐Rad, Hercules, CA, USA) primer sets (Table S1) M145/M146, M149/M151, and Ptq‐74/Ptq‐75 were used for amplification of *Sb*LG1, *Sb*LG2, and Sb*EIF4α* (reference gene), respectively. Transcript copies were quantified via ddPCR using the QX200 Droplet Digital PCR System (Bio‐Rad, Hercules, CA, USA).

### Leaf angle measurements

Leaf angles were assessed under both greenhouse and field environments (2018 & 2020) using a hand‐held protractor as previously described (Jaikumar *et al*., 2021). Angles, relative to vertical, were measured at the blade base, across four plants from each event for each mean tabulation (greenhouse/field plot), on the youngest fully expanded leaf. Blade angles were measured during the late vegetative stage, approximately 5 weeks after planting under field environment and stage 4/5 (flag leaf/boot stage) under greenhouse conditions.

### Field trials and statistics

Field trials were conducted at the University of Illinois‐Champaign campus in 2018 and 2020. Kernels were seed‐coated with Concept III (Syngenta, USA) safener and Mefenoxam systemic fungicide. Row spacing was 76 cm, with approximately 100 kernels per row (3.05 m) in four rows per plot. There were six blocks; each block contained a replicate plot of each of the three events and the wildtype (RTx430). All plots were randomized within a block. Weather data for the May 15–August 31 periods in each year were collected.

Photosynthesis measurements were made in 2018, from July 31 to August 7, when canopy closure was achieved, and the plants were entering booting stage. In 2020, photosynthetic light response curves were measured July 28 to August 4 (at late vegetative stage) and again August 11–17 (at boot stage). An average of two plants per plot was sampled. For each plant, the youngest fully expanded leaf (‘High’) and the lowest fully green leaf (‘Low’) were cut at the leaf base, just before dawn, and were immediately immersed in water and cut again underwater to avoid any cavitation. The samples were then transported to a growth chamber (Conviron: Winnipeg, CA, USA) at 28°C, with 65% humidity and 1000 μmol/m^2^/s Photosynthetic Photon Flux Density (PPFD) to provide a constant environment for measurements. Leaf photosynthetic CO_2_ uptake, stomatal conductance and chlorophyll fluorescence were measured using a portable gas exchange system (LI‐6400 XT; LI‐COR, Lincoln, NE, USA), with [CO_2_] = 400 μL/L, relative humidity between 60% and 70%, and leaf temperature maintained at 33°C in the system's controlled environment cuvette. Transpiration was also monitored in 2020. Leaves were sealed into the cuvette and allowed to acclimate at PPFD = 2000 μmol/m^2^/s until leaf CO_2_ uptake rate (*A*) was stable (less than 3% change over 1 min), typically acclimating for ca. 30 min. Gas exchange and chlorophyll fluorescence were measured at PPFDs of: 2400, 2000, 1600, 1200, 800, 600, 400, 250, 200, 150, 100, 80, 60, 40 and 20 μmol/m^2^/s.

Light response curves for carbon assimilation (*A*) and electron transport (*J*) were constructed by fitting nonrectangular hyperbola functions, using the NL function in STATA 19.1:

(1) $$A=\left[A_{\max}+\Phi_{\mathrm{CO}2,\max,\mathrm{app}}Q\;–\;{\left({\left[A_{\max}+\Phi_{\mathrm{CO}2,\max,\mathrm{app}}Q\right]}^{2}\;–\;4A_{\max}\Phi_{\mathrm{CO}2,\max,\mathrm{app}}\;{\mathrm{Q\theta}}_{A}\right)}^{0.5}\right]/\left(2\theta_{A}\right)\;–\;R_{D}$$

where *A*_max_ is the light‐saturated leaf CO_2_ uptake rate, *Φ*_CO2,max_ is the quantum yield of assimilation under light‐limiting conditions, *Q* is incident PPFD, *θ*_A_ is a convexity parameter describing the rate of transition of light‐limitation to light‐saturation with PPFD and *R*_D_ is light‐adapted respiration excluding photorespiration. For the rate of whole chain electron transport (*J*):

(2) $$J=\left[J_{\max}+\Phi_{J}\;\mathrm{\alpha Q}–{\left({\left[J_{\max}+\Phi_{J}\;\mathrm{\alpha Q}\right]}^{2}–4J_{\max}\Phi_{J}\;{\mathrm{\alpha Q\theta}}_{J}\right)}^{0.5}\right]/\left(2\theta_{J}\right)–J_{0}$$

where *J*_max_ is light‐saturated electron transport rate, *Φ*_J_ is the apparent quantum yield of electron transport under light‐limiting conditions, *Q* is the incident light, *θ*_J_ is a convexity parameter, *J*_0_ is the intercept and α denotes leaf absorptance (measured in 2018).

In 2018, leaf absorptance of leaves used above was measured with an integrating sphere (JAZ Spectrometer, Ocean Optics: Dunedin, FL, USA) coupled with spectrometer operating software (SpectraSuite® Ocean Optics). Canopy light profiles were measured once during the season using a 1‐m line quantum sensor (Decagon Devices: Pullman, WA, USA) between 11:30 a.m. and 12:30 p.m. on 5 August 2018 (at early boot stage). Light measurements were taken above the canopy (*z* = 0) and at depths of 20% (*z* = 0.2), 40% (*z* = 0.4), 60%, 80% and 90% of the distance between the uppermost leaves and the soil surface. From these, extinction coefficients (*k*_ext_) were calculated as follows:

(3) $$Q=Q_{0}\;\exp\;\left(-k_{\mathrm{ext}}z\right)$$

where *z* represents depth into the canopy (starting at *z* = 0 for the top of the canopy), *Q* represents light at a particular depth and *Q*_0_ represents incident light at the top of the canopy.

Concurrently with light profile measurements, leaf area index was measured at the level of the lowest fully green leaf (The lowest fully green leaf occurred about 70% down the canopy, ranging from 55% to 85%, with no difference between events). In 2020, canopy light profiles were measured twice, at late vegetative stage and at the beginning of anthesis. The first measurement was on 2 July 2020, from 11:30 to 12:30 h, as described above for 2018. The second was on 26 August 2020, and involved measuring light profiles periodically over the course of the day, beginning at dawn (ca. 5:00 h) and ending at 19:00 h.

To estimate cumulative photosynthetic carbon assimilation through the canopy, the following procedure was used. The canopy was arbitrarily/subjectively divided into 100 increments, for ease of estimation, according to depth (*Z*), from ground level (*Z* = 1) to the top of the canopy (*Z* = 0). Light level at each value of *Z* was calculated according to Equation 3 above, using the value of *k*_ext_ estimated from the measured canopy light profiles. Using the fits made to Equation 1 above, leaf‐level photosynthetic parameters (e.g. *A*_max_, *Φ*_CO2,max_, *θ*_A_ and *R*_D_) were expressed as a function of incident light level (*Q*) by plotting these parameters, for upper and lower leaves, against the incident light levels for the upper and lower leaves, respectively. Assuming a linear relationship between incident light level and the value of each photosynthetic parameter, values for these parameters were then imputed for each canopy depth *Z*. For each of the 100 depth slices through the canopy, imputed light levels (*Q*) were then combined with the imputed values for *A*_max_, *Φ*_CO2,max_, *θ*_A_ and *R*_D_ to give estimated carbon assimilation at that depth. Estimated assimilation values for the 100 depth slices were then summed, divided by 100 and multiplied by 4 (to account for an assumed leaf area index of 4). In July 2020 these canopy‐level assimilation values were imputed for one time point, at midday (referred to below as *A*_canopy,midday_) since extinction coefficients were only calculated at midday. In August 2020, light profiles through the canopy were measured at multiple time points through the day, starting at dawn and continuing until 7:00 P.M. (Central Daylight Time), at intervals of 1–3 h. Canopy level assimilation values were estimated at each time point and multiplied by the duration of the time interval to the next time point: these were then summed to give an estimate of integrated cumulative photosynthesis over the course of the day (*A*_cumulative_). This procedure was similar to that used by Jaikumar *et al*. (2021), except that rather than considering only the two points of upper and lower canopy it was extended continuously through the whole canopy and over the course of a full day.

Above ground biomass and yield were estimated from a 0.75 m‐long segment of one of the two centre rows from each plot. Immediately prior to anthesis, the panicles of the plants in these segments were bagged, while panicles of the remaining plants were removed in accordance with USDA/APHIS approved standard operating procedure for regulated sorghum. At harvest, plants were separated into grain heads, stems and leaves. Each tissue fraction was weighed, dried at 60 °C for 10 days (until constant mass), and then re‐weighed to obtain dry mass. Grain heads were winnowed to separate grain from chaff. Harvest index (HI) calculations were determined as: Harvested grain/total above‐ground biomass × 100. Days to maturity were not monitored; however, no differences were apparent.

### Crop water use

A tractor‐mounted, customized hydraulic soil corer (Rajurkar *et al*., 2022) was used to install access tubes within crop rows at four locations in each subplot (96 locations per year in total). Soil moisture content was measured at three depths (2–20, 42–60 and 86–104 cm) at each location with a time‐domain reflectometry probe (TRIME‐PICO TDR system, MKO GmbH) as described previously (Ding *et al*., 2022). In 2018, measurements were taken at four time points over an approximately 4‐week period from July 13 to August 18. In 2020 measurements were taken once on June 25 and then again eight times over the 3‐week period from July 27 to August 14.

### Statistical analysis

Plot‐level parameters (leaf angle, extinction coefficient, grain and biomass yield, estimates of canopy‐level carbon assimilation) were analysed using an RCBD ANOVA with transgenic event and block as explanatory variables. In cases where a variable was non‐normally distributed, a Kruskal–Wallis test with event as an explanatory variable was performed instead of the ANOVA. Leaf‐level photosynthetic parameters were analysed using a repeated measures ANOVA, treating event as a between‐subject factor, growth stage and canopy level as within‐subject factors, and individual plots as the subjects. Soil moisture data from each date was averaged across four measurement locations to estimate a plot mean for each depth layer (2–20, 42–60 and 86–104 cm). Plot means were used as input data for a repeated measures ANOVA, with event as a between‐subjects factor, sampling date as a within‐subjects factor and individual plots as the subjects. Additionally, moisture data (at each depth) were analysed separately for each date using a one‐way ANOVA with event as the explanatory variable (if soil moisture data were normally distributed at that date) or alternatively a Kruskal–Wallis test with event as the explanatory variable (if they were not normally distributed).

Analysis was conducted using STATA 19.1 software (StataCorp: College Station, TX USA). If overall ANOVA showed significant effects, *post hoc* testing was carried out. For plot‐level and leaf‐level parameters, transgenic events were compared to the wildtype using Dunnett's test for *m* = 4 groups. For leaf‐level parameters, upper and lower canopy leaves were additionally compared using a paired *t*‐test with the Šidak correction for *m* = 4 comparisons. Normality was assessed by the Shapiro–Wilk test. Following these tests in each of the two individual studies (2018 and 2020), a Fisher's combined test was performed to see if any of the comparisons between events, or the comparisons between lower and upper canopy leaves, yielded effects that were not individually statistically significant in a given year, but were statistically significant when combined across both years. A time‐series of light availability in the lower canopy and projected canopy‐level carbon assimilation at various time points through the day was carried out, wherein pooled datasets collected from the transgenic event plots (combining across events) were compared against the wildtype at each time point using an unpaired unequal‐variance *t*‐test, since individual transgenic events did not differ from each other.

## Author contributions

N.J. conducted field studies and contributed to the drafting of the paper. T.Q. contributed to the molecular characterization of the events. Z.G. assembled the binary vector and assisted in sorghum transformations. N.N. carried out the sorghum transformations. S.J.S. assisted in the sorghum transformations and greenhouse phenotyping. S.M.M. contributed to the field studies and data gathering. A.D.B.L. contributed to the field studies and data analysis. M.G. contributed to the molecular characterizations of the events and the writing of the manuscript. S.P.L. and T.E.C. designed and coordinated the experiments and contributed to the writing of the paper.

## Supporting information


**Table S1** Primer sequences used in RT‐PCR and ddPCR reactions.
**Table S2** Light extinction coefficient (*k*_*ex*t_) through the canopy (field 2020).
**Table S3** Physiological parameters measurements, (*A*_sat_), (*Φ*_CO2,max,app_), (Θ_CO2_), (*R*_D_) and (*Φ*_J_/*Φ*_CO2_) (field 2018).
**Table S4** Physiological parameters measurements, (*A*_sat_), (*Φ*_CO2,max,app_), (Θ_CO2_), (*R*_D_) and (*Φ*_J_/*Φ*_CO2_) (field 2020).
**Table S5** Physiological parameter datasets collected in the 2018 field trial.
**Table S6** Physiological parameter datasets collected in the 2020 field trial.
**Table S7** Soil moisture data 2018 field trial *post hoc* comparison by date.
**Table S8** Soil moisture data 2020 field trial *post hoc* comparison by date.
**Table S9** Water relations and physiological parameter datasets collected in the 2020 field trial.
**Table S10** Weather data for 2018 and 2020 field seasons.

**Figure S1** Diagrammatic view of pPTN1355 T‐DNA element and *Sb*LG1/*Sb*LG2 gene models.
**Figure S2** RT‐PCR and ddPCR assays on leaf blade areas.

## Data Availability

The data that supports the findings of this study are available in the supplementary material of this article.
