# Supplementary material for: Constitutive down‐regulation of liguleless alleles in sorghum drives increased productivity and water use efficiency
Source: Plant Biotechnol J. 2025 Jun 1;23(8):3401–13. doi: 10.1111/pbi.70150 (PMC12310838; doi:10.1111/pbi.70150)
Supplement: Supplementary file 1 — Table S1 Primer sequences used in RT‐PCR and ddPCR reactions. Table S2 Light extinction coefficient (k ext) through the canopy (field 2020). Table S3 Physiological parameters measurements, (A sat), (Φ CO2,max,app), (ΘCO2), (R D) and (Φ J/Φ CO2) (field 2018). Table S4 Physiological parameters measurements, (A sat), (Φ CO2,max,app), (ΘCO2), (R D) and (Φ J/Φ CO2) (field 2020). Table S5 Physiological parameter datasets collected in the 2018 field trial. Table S6 Physiological parameter datasets collected in the 2020 field trial. Table S7 Soil moisture data 2018 field trial post hoc comparison by date. Table S8 Soil moisture data 2020 field trial post hoc comparison by date. Table S9 Water relations and physiological parameter datasets collected in the 2020 field trial. Table S10 Weather data for 2018 and 2020 field seasons. [file PBI-23-3401-s001.docx]

| Table S1: Primer sequences used in RT-PCR and ddPCR reactions | | |
| --- | --- | --- |
| Primer | Annealing target | Sequence |
| P1 | *SbLG*1 | GATGCAAGGCCGATCTCTCC |
| P2 | *SbLG*1 | GCTGCTTTGTTCCCATGTGC |
| P3 | *SbLG*2 | CTTGGAGGATGGAGAGGGCA |
| P4 | *SbLG*2 | TTCTTGATGATCACGCCGGG |
| Primer sets utilized in RTPCR and ddPCR reactions. The location  Each primer annealing locations is depicted in Fig S1. | | |

| Table S2: Light extinction coefficient (*k_ex_*_t_) through the canopy (field 2020) | | | | | | |
| --- | --- | --- | --- | --- | --- | --- |
| **Time** | **ANOVA** | **Kruskal-Wallis** | **RTx430** | **NN567-3-2-1** | **ZG629-6-3a** | **ZG630-5-27d** |
|  | Event | Event |  |  |  |  |
|  | *F*_3,15_ | χ^2^ | --- | --- | --- | --- |
| 5:00 | 1.65 | --- | 1.86 + 0.19 | 1.46 + 0.14 | 1.49 + 0.23 | 1.91 + 0.14 |
| 6:00 | 1.03 | --- | 2.63 + 0.24 | 2.20 + 0.17 | 2.04 + 0.28 | 2.11 + 0.20 |
| 7:00 | 7.99** | --- | 3.36 + 0.11 | 2.22 + 0.21** | 2.47 + 0.17* | 2.56 + 0.09* |
| 8:00 | 4.06* | --- | 2.98 + 0.12 | 2.16 + 0.21 | 2.25 + 0.21 | 2.39 + 0.23 |
| 9:25† | --- | 13.29** | 2.86 + 0.08 | 2.08 + 0.16* | 1.95 + 0.24** | 2.23 + 0.08* |
| 10:50 | 4.31* | --- | 2.50 + 0.16 | 1.83 + 0.22 | 1.78 + 0.28 | 1.71 + 0.14* |
| 12 | 12.44*** | --- | 2.68 + 0.09 | 1.88 + 0.09** | 1.78 + 0.20** | 2.00 + 0.10* |
| 15:00 | 3.01 | --- | 2.86 + 0.22 | 2.27 + 0.27 | 1.98 + 0.33 | 2.31 + 0.17 |
| 17:00 | 0.03 | --- | 3.32 + 0.25 | 3.28 + 0.29 | 3.30 + 0.29 | 3.19 + 0.38 |
| 18:00 | 0.71 | --- | 3.03 + 0.16 | 2.64 + 0.14 | 2.82 + 0.31 | 2.62 + 0.11 |
| Daytime Avg | 12.16*** | --- | 2.88 + 0.04 | 2.07 + 0.18** | 2.04 + 0.14** | 2.16 + 0.06* |
| Light extinction coefficient (*k*_ext_) through the canopy in wildtype sorghum (Texas-430) and three transgenic sorghum events. Data shown are means (+ standard error) and results of statistical significance testing (both analysis of variance and Kruskal-Wallis tests for the significance of ‘event’). Data were collected in mid-August 2020, at 10 points over the course of the day. “Daytime Average” refers to the average of extinction coefficients over the 7:00 – 12:00 period. For *F*-values, symbols ‘*’, ‘**’ and ‘***’ represent statistical significance at α = 0.05, 0.01, and 0.001 respectively; for cell means, these symbols represent statistical significance at α =0.05, 0.01 and 0.001 respectively when each mean is compared to wildtype. Symbol ‘†’ indicates a non-normally distributed variable. Data are from a 2020 field experiment in Savoy, IL. Sample size was *n* = 6 per event. | | | | | | |

| Table S3: Physiological parameters measurements, (*A*_sat_), (Φ_CO2,max,app_), (Θ_CO2_), (*R*_D_) and (Φ_J_ / Φ_CO2_) (field 2018) | | | | | | | |
| --- | --- | --- | --- | --- | --- | --- | --- |
| **Event** | **Stage** | **Level** | ***A*_sat_** | **Φ_CO2,max, app_** | **Θ_CO2_** | ***R*_D_** | **Φ_J_ / Φ_CO2_** |
|  |  |  | **µmol m^-2^ s^-1^** | **---** | **---** | **µmol m^-2^ s^-1^** | **---** |
|  |  |  |  |  |  |  |  |
| Wildtype | Boot | Upper | 55.76 + 1.95 | 0.053 + 0.002* | 0.603+ 0.108 | 3.78 + 0.28 | 5.10 + 0.20 |
| Wildtype | Boot | Lower | 39.67 + 1.95 | 0.041 + 0.002 | 0.520 + 0.086 | 2.20 + 0.30 | 5.53 + 0.26 |
| NN567-3-2-1 | Boot | Upper | 49.86 + 3.96 | 0.049 + 0.002 | 0.378 + 0.187 | 3.80 + 0.17 | 5.14 + 0.11 |
| NN567-3-2-1 | Boot | Lower | 39.73 + 4.21 | 0.041 + 0.002 | 0.394 + 0.091 | 2.34 + 0.19 | 5.99 + 0.58 |
| ZG629-6-3a | Boot | Upper | 46.88 + 5.21 | 0.049 + 0.005 | 0.623 + 0.076 | 4.08 + 0.24 | 4.94 + 0.18 |
| ZG629-6-3a | Boot | Lower | 37.03 + 3.37 | 0.047 + 0.002 | 0.587 + 0.053 | 2.76 + 0.32 | 5.42 + 0.32 |
| ZG630-5-27d | Boot | Upper | 55.64 + 2.23 | 0.052 + 0.004 | 0.338 + 0.080 | 3.97 + 0.28 | 4.63 + 0.24 |
| ZG630-5-27d | Boot | Lower | 39.38 + 2.16 | 0.041 + 0.002 | 0.465 + 0.101 | 2.97 + 0.39 | 5.63 + 0.35 |
|  |  |  |  |  |  |  |  |
| **Source of Variation** | |  | ***F*_19,12_** | ***F*_19,12_** | ***F*_19,12_** | ***F*_19,12_** | ***F*_19,12_** |
| Event | |  | 1.60 | 1.50 | 2.11 | 1.60 | 0.04 |
| Level | |  | 25.99*** | 14.46** | 0.01 | 41.67**** | 14.29** |
| Event x Level | |  | 0.48 | 0.56 | 0.41 | 0.36 | 0.11 |
| Carbon assimilation rate at saturating light (*A*_sat_), apparent quantum yield under light limiting conditions (Φ_CO2,max,app_), convexity of the carbon assimilation vs. light response curve (Θ_CO2_), light adapted respiration (*R*_D_), and ratio of maximal quantum yield of electron transport to quantum yield of assimilation under light limiting conditions (Φ_J_ / Φ_CO2_), in upper canopy and lower canopy leaves of wildtype sorghum (RTx430) and three transgenic sorghum events. Data shown are means (+ standard error) and analysis of variance results. Data were inferred from photosynthetic light response curves measured at boot stage. For *F*-values, symbols ‘*’, ‘**’, ‘***’ and ‘****’ represent statistical significance at α = 0.05, 0.01, 0.001 and 0.0001 respectively. For individual cell means, such symbols represent statistical significance when upper and lower canopy leaves are compared. Symbol ‘†’ represents departure from normality as assessed by the Shapiro-Wilk test. Data are from a 2018 field experiment in Savoy, IL. Sample size was *n* = 4. | | | | | | | |

| Table S4: Physiological parameters measurements, (*A*_sat_), (Φ_CO2,max,app_), (Θ_CO2_), (*R*_D_) and (Φ_J_ / Φ_CO2_) (field 2020) | | | | | | | |
| --- | --- | --- | --- | --- | --- | --- | --- |
| **Event** | **Stage** | **Level** | ***A*_sat_†** | **Φ_CO2,max, app_** | **Θ_CO2_** | ***R*_D_** | **Φ_J_ / Φ_CO2_†** |
|  |  |  | **µmol m^-2^ s^-1^** | **---** | **---** | **µmol m^-2^ s^-1^** | **---** |
| Wildtype | Vegetative | Upper | 53.13 + 5.98 | 0.058 + 0.002 | 0.649 + 0.113 | 3.89 + 0.34 | 5.29 + 0.28 |
| Wildtype | Vegetative | Lower | 46.02 + 4.07 | 0.052 + 0.002 | 0.584 + 0.061 | 3.63 + 0.40 | 5.87 + 0.25 |
| NN567-3-2-1 | Vegetative | Upper | 43.37 + 3.73 | 0.054 + 0.002 | 0.568 + 0.085 | 3.82 + 0.30 | 5.45 + 0.10 |
| NN567-3-2-1 | Vegetative | Lower | 43.98 + 3.68 | 0.053 + 0.002 | 0.660 + 0.050 | 3.14 + 0.42 | 5.77 + 0.20 |
| ZG629-6-3a | Vegetative | Upper | 43.80 + 2.08 | 0.050 + 0.002 | 0.525 + 0.110 | 3.88 + 0.23 | 5.93 + 0.32 |
| ZG629-6-3a | Vegetative | Lower | 43.75 + 2.86 | 0.051 + 0.002 | 0.580 + 0.102 | 3.40 + 0.34 | 5.95 + 0.27 |
| ZG630-5-27d | Vegetative | Upper | 43.67 + 2.99 | 0.050 + 0.002 | 0.553 + 0.094 | 4.04 + 0.39 | 5.92 + 0.15 |
| ZG630-5-27d | Vegetative | Lower | 48.99 + 1.62 | 0.052 + 0.003 | 0.593 + 0.077 | 3.29 + 0.22 | 5.85 + 0.40 |
| Wildtype | Boot | Upper | 46.15 + 4.41** | 0.058 + 0.003* | 0.685 + 0.068 | 4.20 + 0.39* | 5.26 + 0.19 |
| Wildtype | Boot | Lower | 35.69 + 3.25 | 0.048 + 0.002 | 0.588 + 0.084 | 2.51 + 0.36 | 6.02 + 0.20 |
| NN567-3-2-1 | Boot | Upper | 42.43 + 3.56 | 0.056 + 0.003 | 0.684 + 0.068 | 3.66 + 0.44* | 5.36 + 0.15 |
| NN567-3-2-1 | Boot | Lower | 45.32 + 2.26 | 0.056 + 0.004 | 0.443 + 0.067 | 3.17 + 0.45 | 5.54 + 0.34 |
| ZG629-6-3a | Boot | Upper | 49.83 + 5.83 | 0.061 + 0.002* | 0.623 + 0.127 | 4.74 + 0.33** | 5.13 + 0.09 |
| ZG629-6-3a | Boot | Lower | 39.05 + 1.71 | 0.048 + 0.003 | 0.529 + 0.054 | 2.40 + 0.32 | 6.18 + 0.50 |
| ZG630-5-27d | Boot | Upper | 45.48 + 3.14 | 0.058 + 0.003 | 0.632 + 0.138 | 4.51 + 0.39 | 5.09 + 0.10 |
| ZG630-5-27d | Boot | Lower | 34.56 + 2.47 | 0.053 + 0.003 | 0.497 + 0.053 | 2.27 + 0.29 | 6.20 + 0.50 |
| **Source of Variation** | |  | ***F*_75,20_** | ***F*_75,20_** | ***F*_75,20_** | ***F*_75.20_** | ***F*_75,20_** |
| Event | |  | 0.33 | 0.45 | 0.43 | 1.09 | 0.39 |
| Stage | |  | 2.78 | 3.67 | 0.01 | 0.39 | 3.64 |
| Stage x Event | |  | 1.22 | 1.46 | 0.14 | 2.29 | 0.93 |
| Level | |  | 4.53* | 7.21* | 2.09 | 61.32**** | 12.32** |
| Level x Event | |  | 1.54 | 1.44 | 0.13 | 0.12 | 0.92 |
| Stage x Level | |  | 4.39* | 5.02* | 3.08 | 15.52*** | 3.44 |
| Stage x Level x Event | |  | 1.58 | 1.67 | 0.40 | 0.56 | 1.46 |
| Carbon assimilation rate at saturating light (*A*_sat_), apparent quantum yield under light limiting conditions (Φ_CO2,max,app_), convexity of the carbon assimilation vs. light response curve (Θ_CO2_), light adapted respiration (*R*_D_), and ratio of maximal quantum yield of electron transport to quantum yield of assimilation under light limiting conditions (Φ_J_ / Φ_CO2_), in upper canopy and lower canopy leaves of wild-type sorghum (RTx430) and three transgenic sorghum lines. Data shown are means (+ standard error) and analysis of variance results. Data were inferred from photosynthetic light response curves measured both at late vegetative stage and at boot stage. For *F*-values, symbols ‘*’, ‘**’, ‘***’ and ‘****’ represent statistical significance at α = 0.05, 0.01, 0.001 and 0.0001 respectively. For individual cell means, such symbols represent statistical significance when upper and lower canopy leaves are compared. Symbol ‘†’ represents departure from normality as assessed by the Shapiro-Wilk test. Data are from a 2020 field experiment in Savoy, IL. Sample size was *n* = 6. | | | | | | | |

| Table S5: Physiological parameter datasets collected in the 2018 field trial | | | | | | | |
| --- | --- | --- | --- | --- | --- | --- | --- |
| **Event** | **Stage** | **Level** | ***J*_max_** | ***Φ*_J,max, app_** | **Θ_J_†** | ***F*_V_’/*F*_M_’** | ***q*P** |
|  |  |  | **µmol m^-2^ s^-1^** | **---** | **---** | **µmol m^-2^ s^-1^** | **---** |
| Wildtype | Boot | Upper | 180.93 + 0.91* | 0.235 + 0.003 | 0.880 + 0.038 | 0.392 + 0.013 | 0.518 + 0.004 |
| Wildtype | Boot | Lower | 135.44 + 7.78 | 0.206 + 0.007 | 0.841 + 0.078 | 0.384 + 0.007 | 0.407 + 0.007 |
| NN567-3-2-1 | Boot | Upper | 149.92 + 14.81 | 0.209 + 0.016 | 0.854 + 0.069 | 0.372 + 0.015 | 0.465 + 0.027 |
| NN567-3-2-1 | Boot | Lower | 157.63 + 10.12 | 0.218 + 0.004 | 0.730 + 0.098 | 0.391 + 0.013 | 0.451 + 0.035 |
| ZG629-6-3a | Boot | Upper | 150.78 + 6.96 | 0.233 + 0.009 | 0.843 + 0.056 | 0.357 + 0.009 | 0.465 + 0.021 |
| ZG629-6-3a | Boot | Lower | 140.01 + 11.17 | 0.227 + 0.006 | 0.916 + 0.020 | 0.378 + 0.007 | 0.413 + 0.021 |
| ZG630-5-27d | Boot | Upper | 183.86 + 16.71 | 0.229 + 0.011 | 0.797 + 0.092 | 0.394 + 0.008 | 0.435 + 0.040 |
| ZG630-5-27d | Boot | Lower | 147.08 + 9.20 | 0.204 + 0.005 | 0.888 + 0.023 | 0.359 + 0.009 | 0.432 + 0.020 |
| **Source of Variation** | |  | ***F*_19,12_** | ***F*_19,12_** | ***F*_19,12_** | ***F*_19,12_** | ***F*_19,12_** |
| Event | |  | 0.91 | 1.26 | 0.55 | 1.18 | 0.56 |
| Level | |  | 11.99** | 4.05 | 0.00 | 0.00 | 6.94* |
| Event x Level | |  | 3.91* | 0.88 | 1.45 | 3.72* | 1.97 |
| Electron transport rate at saturating light (*J*_max_), apparent quantum yield of electron transport under light limiting conditions (Φ_J,max,app_), convexity of the electron transport vs. light response curve (Θ_J_), maximal potential light-adapted quantum yield of photosystem II under saturating light (*F*_V_^’^/ *F_M_*^’^) and level of photochemical quenching at saturating light (*qP*) in upper canopy and lower canopy leaves of wild-type sorghum (RTx430) and three transgenic sorghum events. Data shown are means (+ standard error) and analysis of variance results. Data were inferred from photosynthetic light response curves measured at boot stage. *F*-values, symbols ‘*’ and ‘**’ represent statistical significance at α = 0.05 and 0.01 respectively. Symbol ‘†’ represents departure from normality as assessed by the Shapiro-Wilk test. Data are from a 2018 field experiment in Savoy, IL. Sample size was *n* = 4. | | | | | | | |

| Table S6: Physiological parameter datasets collected in the 2020 field trial | | | | | | | |
| --- | --- | --- | --- | --- | --- | --- | --- |
| **Event** | **Stage** | **Level** | ***J*_max_** | **Φ_J,max, app_†** | **Θ_J_†** | ***F*_V_^’^/ *F_M_*^’^** | ***qP*** |
|  |  |  | **µmol m^-2^ s^-1^** | **---** | **---** | **---** | **---** |
| Wildtype | Vegetative | Upper | 236.00 + 13.66 | 0.305 + 0.005 | 0.805 + 0.091 | 0.432 + 0.006 | 0.531 + 0.029 |
| Wildtype | Vegetative | Lower | 239.51 + 15.73 | 0.302 + 0.002 | 0.815 + 0.022 | 0.443 + 0.014 | 0.506 + 0.022 |
| NN567-3-2-1 | Vegetative | Upper | 231.17 + 13.75 | 0.294 + 0.090 | 0.782 + 0.060 | 0.426 + 0.011 | 0.529 + 0.024 |
| NN567-3-2-1 | Vegetative | Lower | 230.79 + 14.85 | 0.305 + 0.005 | 0.849 + 0.021 | 0.443 + 0.015 | 0.500 + 0.019 |
| ZG629-6-3a | Vegetative | Upper | 225.21 + 14.02 | 0.294 + 0.007 | 0.748 + 0.066 | 0.419 + 0.008 | 0.519 + 0.028 |
| ZG629-6-3a | Vegetative | Lower | 224.85 + 12.64 | 0.301 + 0.004 | 0.802 + 0.069 | 0.432 + 0.010 | 0.491 + 0.019 |
| ZG630-5-27d | Vegetative | Upper | 244.94 + 9.05 | 0.298 + 0.006 | 0.846 + 0.046 | 0.421 + 0.011 | 0.565 + 0.012 |
| ZG630-5-27d | Vegetative | Lower | 251.92 + 9.46 | 0.296 + 0.005 | 0.807 + 0.032 | 0.438 + 0.006 | 0.537 + 0.016 |
| Wildtype | Boot | Upper | 236.35 + 26.55 | 0.302 + 0.010 | 0.870 + 0.024 | 0.436 + 0.008 | 0.531 + 0.046 |
| Wildtype | Boot | Lower | 190.68 + 12.99 | 0.290 + 0.006 | 0.828 + 0.038 | 0.417 + 0.016 | 0.429 + 0.017 |
| NN567-3-2-1 | Boot | Upper | 221.55 + 19.97 | 0.298 + 0.008 | 0.810 + 0.051 | 0.406 + 0.015 | 0.530 + 0.032 |
| NN567-3-2-1 | Boot | Lower | 224.99 + 12.91 | 0.305 + 0.009 | 0.772 + 0.036 | 0.437 + 0.007 | 0.487 + 0.031 |
| ZG629-6-3a | Boot | Upper | 265.37 + 24.42 | 0.310 + 0.008 | 0.851 + 0.033 | 0.436 + 0.013 | 0.594 + 0.037* |
| ZG629-6-3a | Boot | Lower | 190.11 + 10.47 | 0.290 + 0.005 | 0.814 + 0.047 | 0.411 + 0.012 | 0.438 + 0.018 |
| ZG630-5-27d | Boot | Upper | 227.86 + 19.32 | 0.294 + 0.012 | 0.833 + 0.072 | 0.412 + 0.015 | 0.537 + 0.036 |
| ZG630-5-27d | Boot | Lower | 198.99 + 9.68 | 0.293 + 0.005 | 0.716 + 0.082 | 0.434 + 0.012 | 0.402 + 0.030 |
| **Source of Variation** | |  | ***F*_75,20_** | ***F*_75,20_** | ***F*_75,20_** | ***F*_75,20_** | ***F*_75,20_** |
| Event | |  | 0.00 | 0.09 | 0.35 | 0.26 | 0.15 |
| Stage | |  | 4.89* | 0.00 | 0.03 | 1.50 | 5.61* |
| Stage x Event | |  | 1.50 | 0.58 | 0.78 | 0.14 | 2.83 |
| Level | |  | 5.71* | 0.00 | 0.38 | 2.54 | 18.29*** |
| Level x Event | |  | 1.01 | 1.03 | 0.55 | 2.44 | 0.60 |
| Stage x Level | |  | 13.19** | 0.87 | 2.35 | 1.15 | 13.28** |
| Stage x Level x Event | |  | 1.97 | 1.42 | 0.04 | 1.26 | 1.24 |
| Electron transport rate at saturating light (*J*_max_), apparent quantum yield of electron transport under light limiting conditions (Φ_J,max,app_), convexity of the electron transport vs. light response curve (Θ_J_), maximal potential light-adapted quantum yield of photosystem II under saturating light (*F*_V_^’^/ *F_M_*^’^) and level of photochemical quenching at saturating light (*qP*) in upper canopy and lower canopy leaves of wildtype sorghum (RTx430) and three transgenic sorghum lines. Data shown are means (+ standard error) and analysis of variance results. Data were inferred from photosynthetic light response curves measured both at late vegetative stage and at boot stage. For *F*-values, symbols ‘*’, ‘**’ and ‘***’ represent statistical significance at α = 0.05, 0.01 and 0.001 respectively. Symbol ‘†’ represents departure from normality as assessed by the Shapiro-Wilk test. Data are from a 2020 field experiment in Savoy, IL. Sample size was *n* = 6. | | | | | | | |

| Table S7: Soil moisture data 2018 field trial *post hoc* comparison by date | | | | | |
| --- | --- | --- | --- | --- | --- |
| **Event** | **Date** | **Moisture% (0.5 m)** | **Moisture% (1.0 m)** | **Moisture% (1.5)** |  |
| Wildtype | Jul 13 | 34.8 + 1.3 | 37.5 + 0.8 | 34.1 + 1.7 |  |
| NN567-3-2-1 | Jul 13 | 35.9 + 1.4 | 36.3 + 2.1 | 34.7 + 2.8 |  |
| ZG629-6-3a | Jul 13 | 35.2 + 1.3 | 36.9 + 3.3 | 34.2 + 2.5 |  |
| ZG630-5-27d | Jul 13 | 36.3 + 0.9 | 37.8 + 0.6 | 33.9 + 2.0 |  |
| *F*_3,9_ | Jul 13 | 0.57 | 0.17 | 0.03 |  |
| Wildtype | Jul 18 | 42.7 + 2.6 | 42.0 + 2.0 | 35.9 + 3.3 |  |
| NN567-3-2-1 | Jul 18 | 41.6 + 1.4 | 41.2 + 3.0 | 37.6 + 2.5 |  |
| ZG629-6-3a | Jul 18 | 39.9 + 1.1 | 40.1 + 1.9 | 34.3 + 2.0 |  |
| ZG630-5-27d | Jul 18 | 42.1 + 0.9 | 39.4 + 1.6 | 33.6 + 2.6 |  |
| *F*_3,9_ | Jul 18 | 0.81 | 0.34 | 0.56 |  |
| Wildtype | Jul 28 | 35.9 + 2.6 | 35.9 + 0.8 | 33.4 + 1.8 |  |
| NN567-3-2-1 | Jul 28 | 35.0 + 2.7 | 35.2 + 1.8 | 36.4 + 2.9 |  |
| ZG629-6-3a | Jul 28 | 36.4 + 2.1 | 37.2 + 2.1 | 36.2 + 1.9 |  |
| ZG630-5-27d | Jul 28 | 36.1 + 1.8 | 35.2 + 1.0 | 34.6 + 2.7 |  |
| *F*_3,9_ | Jul 28 | 0.27 | 0.70 | 0.34 |  |
| Wildtype | Aug 8 | 37.0 + 1.0 | 39.6 + 1.0 | 36.6 + 2.1 |  |
| NN567-3-2-1 | Aug 8 | 37.6 + 0.9 | 36.6 + 1.7 | 37.4 + 2.8 |  |
| ZG629-6-3a | Aug 8 | 37.0 + 0.8 | 37.3 + 1.5 | 34.1 + 1.9 |  |
| ZG630-5-27d | Aug 8 | 40.8 + 1.0 | 38.0 + 1.0 | 35.0 + 2.7 |  |
| *F*_3,9_ | Aug 8 | 3.13 | 1.20 | 0.53 |  |
| **Variable** |  | ***F*_27,36_** | ***F*_27,36_** | ***F*_27,36_** |  |
| Event |  | 0.27 | 0.21 | 0.20 |  |
| Date |  | 33.32**** | 20.64**** | 1.18 |  |
| Date x Event |  | 1.21 | 0.88 | 0.86 |  |
| Means (+ standard error) for soil moisture at three soil depths in wildtype sorghum (RTx430) and three transgenic sorghum events. For *F*-values, symbols ‘*’, ‘**’, ‘***’ and ‘****’ represent statistical significance at α = 0.05, 0.01, 0.001 and 0.0001 respectively. Analysis of variance testing was done at each individual date to test whether events were significantly different. Data are from a 2018 field experiment in Savoy, IL. Sample size was *n* = 4. | | | | | |

| Table S8: Soil moisture data 2020 field trial *post hoc* comparison by date | | | | |  |
| --- | --- | --- | --- | --- | --- |
| **Event** | **Date** | **Moisture% (0.5m)** | **Moisture% (1.0m)** | **Moisture%(1.5m)** | |
| Wildtype | Jun 25 | 30.5 + 0.3 | 30.5 + 0.3 | 29.5 + 0.7 | |
| NN567-3-2-1 | Jun 25 | 30.6 + 0.3 | 31.6 + 0.3 | 31.0 + 0.1 | |
| ZG629-6-3a | Jun 25 | 29.8 + 0.3 | 29.9 + 0.5 | 29.2 + 0.4 | |
| ZG630-5-27d | Jun 25 | 30.2 + 0.4 | 30.6 + 0.3 | 30.2 + 0.4 | |
| *F*_3,9_ | Jun 25 | 1.07 | 2.07 | 2.13 | |
| Wildtype | Jul 27 | 28.9 + 1.0 | 41.9 + 2.6 | 49.5 + 3.0 | |
| NN567-3-2-1 | Jul 27 | 29.6 + 0.6 | 44.6 + 1.6 | 48.8 + 2.2 | |
| ZG629-6-3a | Jul 27 | 28.7 + 0.8 | 41.9 + 2.8 | 44.6 + 3.3 | |
| ZG630-5-27d | Jul 27 | 27.9 + 0.6 | 43.3 + 2.6 | 47.5 + 3.1 | |
| *F*_3,15_ | Jul 27 | 0.68 | 0.27 | 0.53 | |
| Wildtype | Jul 31 | 28.3 + 0.7 | 36.2 + 3.0 | 49.2 + 4.3 | |
| NN567-3-2-1 | Jul 31 | 29.4 + 0.8 | 36.6 + 1.5 | 50.0 + 2.5 | |
| ZG629-6-3a | Jul 31 | 27.7 + 0.7 | 33.6 + 1.5 | 44.9 + 3.4 | |
| ZG630-5-27d | Jul 31 | 27.5 + 1.0 | 35.2 + 1.7 | 49.6 + 2.9 | |
| *F*_3,15_ | Jul 31 | 1.06 | 0.34 | 0.48 | |
| Wildtype | Aug 2 | 41.4 + 3.3 | 38.4 + 3.2 | 42.7 + 5.1 | |
| NN567-3-2-1 | Aug 2 | 45.0 + 2.2 | 35.7 + 1.1 | 41.7 + 2.1 | |
| ZG629-6-3a | Aug 2 | 41.5 + 2.6 | 35.0 + 2.1 | 41.6 + 4.2 | |
| ZG630-5-27d | Aug 2 | 47.8 + 2.1 | 34.4 + 0.7 | 40.4 + 2.9 | |
| *F*_3,15_ | Aug 2 | 1.90 | --- | --- | |
| Kruskal-Wallis χ^2^ | Aug 2 | --- | 0.61 | 0.75 | |
| Wildtype | Aug 4 | 33.1 + 0.7 | 38.9 + 2.3 | 47.0 + 3.5 | |
| NN567-3-2-1 | Aug 4 | 33.8 + 0.7 | 38.9 + 1.5 | 47.9 + 2.6 | |
| ZG629-6-3a | Aug 4 | 32.5 + 0.9 | 37.6 + 1.7 | 44.3 + 2.8 | |
| ZG630-5-27d | Aug 4 | 33.2 + 0.7 | 40.8 + 2.0 | 47.8 + 2.8 | |
| *F*_3,15_ | Aug 4 | 0.44 | 0.40 | 0.27 | |
| Wildtype | Aug 6 | 30.8 + 0.4 | 37.6 + 2.6 | 44.7 + 4.0 | |
| NN567-3-2-1 | Aug 6 | 31.0 + 0.6 | 36.2 + 2.0 | 44.8 + 2.5 | |
| ZG629-6-3a | Aug 6 | 30.2 + 0.9 | 35.4 + 1.2 | 38.8 + 2.4 | |
| ZG630-5-27d | Aug 6 | 29.8 + 0.7 | 36.4 + 2.0 | 42.9 + 3.5 | |
| *F*_3,15_ | Aug 6 | 0.62 | 0.17 | 0.76 | |
| Wildtype | Aug 8 | 28.7 + 0.5 | 34.1 + 1.5 | 44.7 + 4.0 | |
| NN567-3-2-1 | Aug 8 | 29.4 + 0.3 | 35.3 + 1.4 | 44.9 + 2.7 | |
| ZG629-6-3a | Aug 8 | 28.7 + 0.7 | 32.7 + 1.6 | 40.1 + 2.2 | |
| ZG630-5-27d | Aug 8 | 28.5 + 0.8 | 33.8 + 1.7 | 43.9 + 2.6 | |
| *F*_3,15_ | Aug 8 | 0.41 | 0.34 | 0.48 | |
| Wildtype | Aug 11 | 28.5 + 0.6 | 33.5 + 1.8 | 42.9 + 4.3 | |
| NN567-3-2-1 | Aug 11 | 27.8 + 1.6 | 33.9 + 2.2 | 42.2 + 3.8 | |
| ZG629-6-3a | Aug 11 | 28.7 + 0.7 | 33.0 + 1.5 | 38.8 + 2.1 | |
| ZG630-5-27d | Aug 11 | 28.4 + 1.0 | 33.3 + 1.7 | 44.5 + 3.0 | |
| *F*_3,15_ | Aug 11 | --- | 0.04 | 0.48 | |
| Kruskal-Wallis χ^2^ | Aug 11 | 0.05 | --- | --- | |
| Wildtype | Aug 14 | 26.0 + 0.6 | 29.8 + 0.7 | 37.4 + 3.1 | |
| NN567-3-2-1 | Aug 14 | 26.5 + 0.5 | 30.4 + 1.6 | 37.5 + 3.2 | |
| ZG629-6-3a | Aug 14 | 26.0 + 0.2 | 29.1 + 1.3 | 34.4 + 1.9 | |
| ZG630-5-27d | Aug 14 | 26.3 + 0.9 | 31.6 + 1.8 | 38.6 + 1.7 | |
| *F*_3,15_ | Aug 14 | 0.20 | 0.56 | 0.55 | |
| **Variable** |  | ***F*_51,140_** | ***F*_51,140_** | ***F*_51,140_** | |
| Event |  | 0.53 | 0.22 | 0.41 | |
| Date |  | 116.02**** | 50.41**** | 33.72**** | |
| Date x Event |  | 1.28 | 0.84 | 0.75 | |
| Means (+ standard error) for soil moisture at three soil depths in wildtype sorghum (RTx430) and three transgenic sorghum events. For *F*-values, symbols ‘*’, ‘**’, ‘***’ and ‘****’ represent statistical significance at α = 0.05, 0.01, 0.001 and 0.0001 respectively. Analysis of variance testing was done at each individual date to test whether events were significantly different. In cases where moisture was non-normally distributed, Kruskal-Wallis testing was done as an alternative to ANOVA. Data are from a 2020 field experiment in Savoy, IL. Sample size was *n* = 6 per event for all sampling dates except June 25, where sample size was *n* = 4. | | | | | |

| Table S9: Water relations and physiological parameter datasets collected in the 2020 field trial | | | | | | | | |
| --- | --- | --- | --- | --- | --- | --- | --- | --- |
| **Event** | **Stage** | **Level** | ***G*_S,max_** | **Φ_G,app_** | ***Θ*_G_** | ***IWUE*_ambient_** | ***E*_ambient_** | ***WUE*_inst,ambient_** |
|  |  |  | mmol m^-2^ s^-1^ | µmol µmol^-1^ | --- | µmol mol^-1^ | mmol m^-2^ s^-1^ | µmol mmol^-1^ |
| Wildtype | Vegetative | Upper | 287 + 46 | 350 + 28 | 0.546 + 0.139 | 151 + 7 | 6.78 + 0.97 | 5.53 + 0.34 |
| Wildtype | Vegetative | Lower | 287 + 21 | 320 + 52 | 0.464 + 0.140 | 139 + 10 | 4.21 + 0.37 | 4.95 + 0.52 |
| NN567-3-2-1 | Vegetative | Upper | 251 + 19 | 359 + 35 | 0.545 + 0.088 | 156 + 9 | 6.13 + 0.46 | 5.63 + 0.54 |
| NN567-3-2-1 | Vegetative | Lower | 268 + 27 | 331 + 43 | 0.561 + 0.146 | 148 + 12 | 4.91 + 0.73 | 5.78 + 0.57 |
| ZG629-6-3a | Vegetative | Upper | 240 + 28 | 277 + 35 | 0.495 + 0.128 | 171 + 8 | 5.73 + 0.56 | 6.08 + 0.46 |
| ZG629-6-3a | Vegetative | Lower | 284 + 47 | 290 + 19 | 0.589 + 0.137 | 152 + 3 | 4.62 + 0.49 | 5.82 + 0.63 |
| ZG630-5-27d | Vegetative | Upper | 255 + 26 | 282 + 55 | 0.359 + 0.141 | 161 + 5 | 6.48 + 0.54 | 5.24 + 0.34 |
| ZG63-5-27d | Vegetative | Lower | 384 + 69 | 326 + 24 | 0.438 + 0.106 | 147 + 3 | 5.11 + 0.32 | 5.83 + 0.24 |
|  |  |  |  |  |  |  |  |  |
| Wildtype | Boot | Upper | 301 + 62 | 516 + 136 | 0.452 + 0.148 | 146 + 13 | 6.79 + 0.80 | 5.56 + 0.21 |
| Wildtype | Boot | Lower | 241 + 28 | 464 + 121 | 0.301 + 0.164 | 129 + 12 | 2.55 + 0.37 | 7.75 + 1.10 |
| NN567-3-2-1 | Boot | Upper | 284 + 58 | 393 + 45 | 0.542 + 0.102 | 143 + 11 | 6.36 + 0.56 | 5.24 + 0.39 |
| NN567-3-2-1 | Boot | Lower | 292 + 20 | 342 + 23 | 0.345 + 0.053 | 157 + 6 | 2.90 + 0.24 | 9.28 + 0.76 |
| ZG629-6-3a | Boot | Upper | 434 + 91 | 354 + 70 | 0.401 + 0.148 | 150 + 25 | 7.42 + 0.74 | 5.17 + 0.27 |
| ZG629-6-3a | Boot | Lower | 234 + 22 | 287 + 32 | 0.301 + 0.118 | 171 + 8 | 3.04 + 0.24 | 8.02 + 0.40 |
| ZG630-5-27d | Boot | Upper | 273 + 42 | 403 + 67 | 0.446 + 0.114 | 157 + 13 | 6.57 + 0.97 | 5.95 + 0.62 |
| ZG630-5-27d | Boot | Lower | 229 + 23 | 467 + 139 | 0.264 + 0.109 | 138 + 7 | 2.90 + 0.30 | 8.16 + 0.55 |
| **Source of Variation** | |  | ***F*_75,20_** | ***F*_75,20_** | ***F*_75,20_** | ***F*_75,20_** | ***F*_75,20_** | ***F*_75,20_** |
| Event | |  | 0.25 | 1.84 | 0.21 | 2.13 | 0.08 | 0.40 |
| Stage | |  | 0.03 | 6.48* | 2.81 | 1.02 | 4.49* | 29.44**** |
| Stage x Event | |  | 1.63 | 0.96 | 0.65 | 0.14 | 0.61 | 0.83 |
| Level | |  | 0.47 | 0.13 | 0.45 | 1.52 | 117.03**** | 37.20**** |
| Level x Event | |  | 1.77 | 0.36 | 0.27 | 0.88 | 0.84 | 1.33 |
| Stage x Level | |  | 6.32* | 0.15 | 1.91 | 1.14 | 20.39*** | 32.18**** |
| Stage x Level x Event | |  | 1.24 | 0.09 | 0.11 | 0.85 | 0.40 | 0.89 |
| Means (+ standard error) and analysis of variance results for stomatal conductance at saturating light (*G*_S,max_), quantum yield of stomatal conductance (Φ_G,app_**)**, convexity of stomatal conductance vs. light response curve (θ_G_), estimated intrinsic water use efficiency under ambient light conditions (*IWUE*_ambient_), estimated transpiration under ambient light conditions (*E*_ambient_) and estimated instantaneous water use efficiency under ambient light conditions (*WUE*_inst,ambient_, or *A*_ambient_/*E*_ambient_) in upper canopy and lower canopy leaves. The estimates of *G*_S,max_, Φ_G,app_ and θ_G_ were inferred from photosynthetic light response curves measured both at late vegetative and at boot stage. For transpiration, intrinsic water use efficiency and instantaneous water use efficiency under ambient light, data were estimated from transpiration vs. light response curves combined with light profiles through the canopy. For *F*-values, symbols ‘*’ and ‘**’ represent statistical significance at α = 0.05 and 0.01 respectively. Data are from a 2020 field experiment in Savoy, IL. Sample size was *n* = 6. | | | | | | | | |

| Table S10: Weather data for 2018 and 2020 field seasons | | | | |
| --- | --- | --- | --- | --- |
| **Month** | **Ave Temp (°C)** | **<15°C** | **>32°C** | **Precipitation (mm)** |
| May 2018 | 18.8 | 2 | 4 | 52 |
| June 2018 | 23.2 | 4 | 4 | 171 |
| July 2018 | 22.7 | 4 | 1 | 150 |
| August 2018 | 23.3 | 4 | 3 | 80 |
| May 2020 | 15.5 | 11 | 0 | 104 |
| June 2020 | 23.2 | 8 | 5 | 74 |
| July 2020 | 24.9 | 0 | 10 | 96 |
| August 2020 | 22.3 | 3 | 7 | 40 |
| Ave Temp column reflects the average temperature for that month/year. <15°C  and >32°C columns indicate number of days in that month/year temperatures  were below 15°C or above 32°C in the given month/year. Precipitation column  is the total rain fall in that month/year. | | | | |
