# Supplementary material for: Constitutive down‐regulation of liguleless alleles in sorghum drives increased productivity and water use efficiency
Source: Plant Biotechnol J. 2025 Jun 1;23(8):3401–13. doi: 10.1111/pbi.70150 (PMC12310838; doi:10.1111/pbi.70150)
Supplement: Supplementary file 2 — Figure S1 Diagrammatic view of pPTN1355 T‐DNA element and SbLG1/SbLG2 gene models. Figure S2 RT‐PCR and ddPCR assays on leaf blade areas. [file PBI-23-3401-s002.docx]

**Fig. S1**. Diagrammatic view of pPTN1355 T-DNA element and *Sb*LG1/*Sb*LG2 gene models


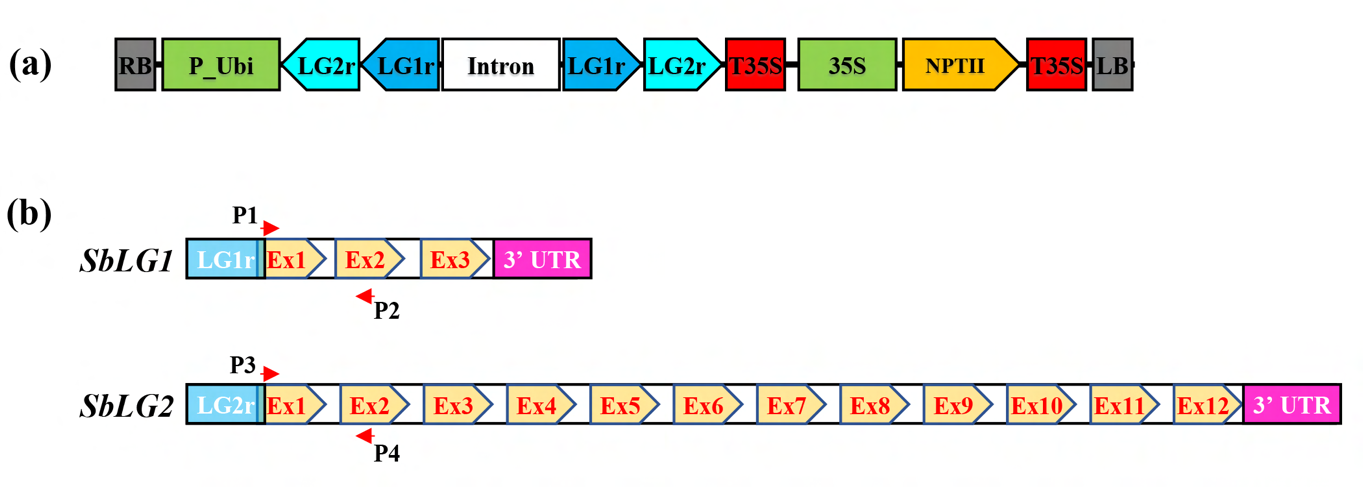


**Fig. S1a**. Diagrammatic view of pPTN1355 T-DNA element. RB and LB refer to right and left border elements, respectively. P-Ubi- maize ubiquitin promoter. 35S and T35S- CaMV promoter and terminator, respectively. Intron- second intron of Arabidopsis gene model At4G02840. LG2/LG1- are the 250bp regions that make-up one hair-pin arm. **Fig. S1b**. Diagrammatic view of *Sb*LG1 (SbiRTX430.06G264300.1) and *Sb*LG2 (SbiRTX430.03G392300.1) gene models. P1, P2, P3, P4 highlight annealing sites of primer sets utilized in RT-PCR and ddPCR assays.

**Fig.S2**. RT-PCR and ddPCR assays on leaf blade areas


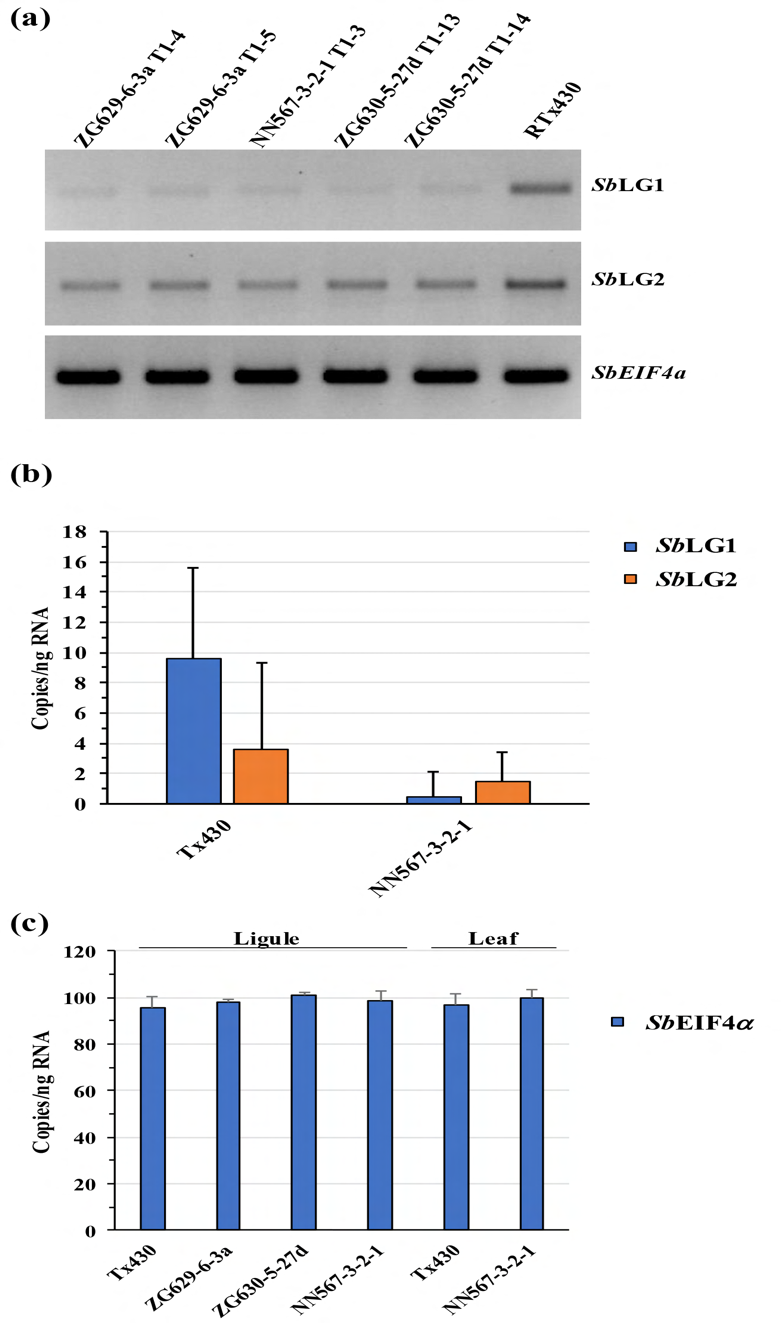


Fig.S2a. RT-PCR on RTx430 (control) and the 3 respective *Sb*LG1/*Sb*LG2 hair-pin events. Two T_1_ individuals from ZG629-6-3a; two T_1_ individuals from ZG630-5-27a, one T1 individuals from NN567-3-2-. EIF4α (reference gene). Fig.S2b. ddPCR results (copies per ng RNA) on RTx430 (control) and the *Sb*LG1/*Sb*LG2 hair-pin event NN567-3-2-1. Fig.S2c. ddPCR results (copies per ng RNA) on RTx430 and SbLG1/SbLG2 hair pin events from ligule region and leaf blade proper of reference gene EIF4α.
